# Genetic structuring and invasion status of the perennial *Ambrosia psilostachya* (*Asteraceae*) in Europe

**DOI:** 10.1038/s41598-023-30377-6

**Published:** 2023-03-06

**Authors:** Gerhard Karrer, Rea Maria Hall, Valérie Le Corre, Matthias Kropf

**Affiliations:** 1 Institute of Botany, University of Natural Resources and Life Sciences Vienna, Vienna, Austria; 2 INRAE, UMR1347 Agroécologie, Dijon, France; 3 Institute of Integrative Nature Conservation Research, University of Natural Resources and Life Sciences Vienna, Vienna, Austria

**Keywords:** Ecology, Genetics, Plant sciences

## Abstract

The perennial western ragweed (*Ambrosia psilostachya* DC.) arrived from North America to Europe in the late nineteenth century and behaves invasive in its non-native range. Due to its efficient vegetative propagation via root suckers, *A. psilostachya* got naturalized in major parts of Europe forming extensive populations in Mediterranean coastal areas. The invasion history, the spreading process, the relationships among the populations as well as population structuring is not yet explored. This paper aims to give first insights into the population genetics of *A. psilostachya* in its non-native European range based on 60 sampled populations and 15 Simple Sequence Repeats (SSR). By AMOVA analysis we detected 10.4% of genetic variation occurring among (pre-defined) regions. These regions represent important harbors for trading goods from America to Europe that might have served as source for founder populations. Bayesian Clustering revealed that spatial distribution of genetic variation of populations is best explained by six groups, mainly corresponding to regions around important harbors. As northern populations show high degrees of clonality and lowest levels of within-population genetic diversity (mean *H*_*o*_ = 0.40 ± 0.09), they could preserve the initial genetic variation levels by long-lived clonal genets. In Mediterranean populations *A. psilostachya* expanded to millions of shoots. Some of those were obviously spread by sea current along the coast to new sites, where they initiated populations characterized by a lower genetic diversity. For the future, the invasion history in Europe might get clearer after consideration of North American source populations of western ragweed.

## Introduction

Invasive species increase the local species richness^1^ but can also threaten native biodiversity by competition at various levels^2^. Beyond biodiversity threats, invasive plants could also reduce yield from agricultural fields^3^ and directly affect human health^4^. The latter holds for species of the genus *Ambrosia* that produce highly allergenic pollen^5^. Particularly, *A. artemisiifolia* L. (common ragweed) is well-documented to cause health problems like allergic rhinitis (hay fever) and asthma in its native range (North America^6^) as well as in invaded continents like Europe, Asia, Australia, and Africa^7^. All *Ambrosia* species are wind-pollinated and produce high numbers of pollen during summer^8^. Common ragweed is most striking for its release of allergic pollen^5,9^, and some other species (i.e. *A. trifida* L.*, A. psilostachya* DC., *A. confertiflora* DC.) are also known to cause allergic diseases^10^. At the moment, five non-native *Ambrosia* species are documented from Europe^11^: *A. artemisiifolia, A. psilostachya, A. tenuifolia* Schreb.*, A. trifida,* and *A. tomentosa* Nutt. A sixth species, *A. maritima* L., is supposed to be native to the Old World^8,12,13^. Only one of the alien ragweed species is widespread throughout temperate Europe (i.e. *A. artemisiifolia*^12,14^), continuously enlarging its distribution area towards northern regions–possibly favored by climate warming^15–17^. Western (= perennial) ragweed, *A. psilostachya*, is documented from several continents^18^ and occurs in many European countries^19^ behaving invasive in some regions^3,16,20,21^. It is treated taxonomically as conspecific to some other taxa from North America, i.e. to *A. coronopifolia* Torrey & Gray^6,8^. In its native range in Northern Mexico, USA (in 42 out of 50 federal states^6^) and all southern states of Canada^6^, *A. psilostachya* prefers open vegetation on sandy soils, i.e. prairie grasslands, but also alluvial areas and ruderal places^6,8,18,21^. This perennial herb is co-dominant to other grassland herbs and grasses, specifically in the Great Plains^22,23^, but prefers ruderal places towards the Great Lakes area and South-eastern Canada^24^. Naturalization of western ragweed happened even over a wider geographical range than in common ragweed due to the formation of root sprouts that survive cold winters belowground and allow clonal growth. An extensive review of specimens in European herbarium collections by the first author uncovered about 10% misidentifications by the collectors. Problems with identification resulted mostly from less accurate determination keys in floras like those by Hansen^25^, Sell & Murrell^26^, or Pignatti^27^. *A. psilostachya* is perennial with an extensive belowground root and shoot system^8,11^. Extensive populations are known from the Mediterranean coast of Spain^28^, France^3^, and Italy^11,21,29^ but also from the easternmost continental part of Europe (Russia^20^). *A. psilostachya* populations along the Northern European coastlines and inland sand habitats in Central Europe (Germany and Poland) are scattered. Perennation by root sprouts allows this invasive alien to survive in stabile populations on sandy substrates even in rather northern climates like Sweden (Falkenberg, 50 km S of Gothenburg, G. Karrer, pers. obs.) or north-western England (Birkdale near Southport, P. Ashton, pers. comm.). In such regions the annual *A. artemisiifolia* cannot naturalize due to failure of seed production^12^. The northernmost ephemeral finding of western ragweed is from Kurtto, Finland (pers. herbarium collection J. Särkkä, 2007, https://laji.fi/en/taxon/MX.42782).

However, invasion history of *A. psilostachya* to Europe is not well-documented; the first specimens were reported from Great Britain in 1880^30^, France in 1891^31^, Germany in 1894 (herbarium Leiden: L 3,003,673), Italy in 1924 (herbarium Pisa), Spain in 1947^32^, and Russia in 1934 (specimen in the herbarium of Ufa). In Northern Africa, the first specimen was documented in 1918 from Algiers (herbarium Leiden: L 3,004,391). It is assumed that *A. psilostachya* arrived to Europe by traded crops^21^, i.e. potatoes from the Americas may have been contaminated by root fragments of western ragweed. No data is available about the likely source regions in America for the introductions to Europe. In Southern Europe, the invasion or expansion process is ongoing. In contrast, towards the north, there is no documentation about any increase of population sizes or further spread from initial places of introduction. Several accidental introductions in Central or Northern Europe (Austria, Hungary, Czech Republic, Denmark, Estonia, Finland, Norway) turned out to be ephemeral^19,33^.

Important biological traits of *A. psilostachya* like the prevailing asexual reproduction by root sprouts were documented mostly from its native range in North America^31,34^. Wagner & Beals^24^ described a rapid increase of single shoot spatial expansion up to 2 m^2^ within the second year of establishment. Consequently, clonal growth might result in phenotypically uniform populations. In the native as well as in the (European) invasive range, *A. psilostachya* regenerates rarely from seeds^3,35^. From Europe there is only few data available about biology and ecological preferences. E.g. Fried et al.^3^ reported biological traits of French *A. psilostachya* seed lots and established populations’ habitat as well as soil preferences. Obviously, belowground spreading rates are quite high what can be deduced from the huge dominant stands in Italian coastal dunes^21^. Djemaa^35^ documented germination rates of only 3% in French seed lots. This can be due to unfavorable pollination conditions or to population genetics (e.g. inbreeding depression).

While population genetics and invasion history of *A. artemisiifolia* is relatively well analyzed^36–49^, no research group has yet studied the population genetics of the clonal *A. psilostachya* neither in the native nor in its invasive range. No detailed taxonomic study was performed on this difficult diploid-polyploid complex since the monograph by Payne^13^. Greatly varying chromosome counts at diploid, tetraploid, hexaploid and octoploid levels are documented from North American populations of *A. psilostachya*^50–52^. In addition, reports from North America showed considerable morphological^13,24^ and phytochemical variation^51^ of *A. psilostachya*. But none of the authors linked this trait variation to ploidy levels.

In case of clonal species like *A. psilostachya*, one has to adapt methods when analyzing population genetic structure^53,54^. By setting the ranges for screening multilocus genotypes and adjusting the admixture models^55^, the outcome of the analyses (e.g. what is accepted as clonal offspring) may be influenced. Newly introduced populations of partially clonal plants tend to sustain the initial within-population genetic structure^56^. Bottleneck effects as well as gene flow have less or even almost no consequence on the population genetics of clonal species^57^.

Meyer et al.^45^ developed nuclear SSR-markers (Simple Sequence Repeats) for *A. artemisiifolia* and tested them in a population of *A. psilostachya*. These authors claimed that most of these markers are useful in describing western ragweed population genetics. In the present paper, we utilized these SSR markers to dissect the population genetic structure of *A. psilostachya* in Europe, providing insights into the genetic differences considering varying population sizes and local introduction histories.

We therefore aim to answer the following questions: (1) How strong is the genetic differentiation of *A. psilostachya* populations in Europe? (2) Is there an effect of different degrees of clonality within the populations on the invasion process and success across Europe? (3) How are genetic diversity patterns correlated to presumed age of populations (i.e. arrival date) and/or population sizes? (4) Are there different possible centers of introduction detectable based on the genetic structuring? (5) How much do population genetic characteristics differ between the two successful *Ambrosia* invaders, i.e. the annual *A. artemisiifolia* and the perennial *A. psilostachya*?

## Results

Based on extensive studies on literature and herbarium collections reviewed by the first author, we sampled 60 populations from Middle Sweden to Southern Italy and from Spain to Croatia. From 1005 analyzed individuals representing 60 European populations, 50 individual samples were excluded from further analysis due to unreliable genotyping fragment patterns and/or missing data. Therefore, running 955 remaining individuals, we could avoid that some R applications treat missing states as novel alleles which would lead to bias in our results^58^. The frequencies of null alleles estimated over all populations ranged between 0.01 and 0.27 (mean over all loci: 0.09 with original as well as clone corrected data). Significant linkage disequilibrium was detected among loci as well as among populations and regions.

### MLG diversity and clonality

The 15 SSR markers utilized showed an average of 2.56 ± 0.74 alleles per locus (= Na, ranging from 1.45 to 3.98). After clone correction, we gained 2.45 ± 0.63 alleles per locus (Table 1). Based on a minimum genetic dissimilarity threshold of 0.5, we identified 792 unique multilocus genotypes (MLGs) among the 955 originally analyzed individuals. Based on Bonin et al.^59^, our 15 loci should be adequate to detect a reliable number of unique MLGs. When comparing the genetic parameters calculated with and without clone correction (Supplementary Table S1) it is evident that there are hardly serious differences. Consequently, we discuss preferably the results of the analyses based on clone corrected (cc) data.

**Table 1:** Population descriptors and genetic diversity of European populations of *Ambrosia psilostachya*: region (main harbors, based on introduction history), size (number of ramets per population), age (maximum age based on the introduction history in the respective region); genetic diversity parameters (calculations based on the clone corrected data = ‘cc’); n number of ramets sampled, N number of ramets genotyped, G number of unique multilocus genotypes, G/N genet/ramet ratio, H Shannon diversity index (based on N), E genotypic Evenness, Na allelic richness (cc), *H*_*o*_ observed heterozygosity (cc), *H*_*e*_ expected heterozygosity (cc), *F*_is_ inbreeding coefficient (cc).

ID	Region	Size	Age	n	N	G	G/N	H	E	Na cc	*Ho* cc	*He* cc	*F*is cc
Psi-03	Montpellier	100	120	10	10	10	1.00	2.30	1.00	3.31	0.59	0.58	0.05
Psi-04	Montpellier	5000	120	8	7	7	1.00	1.95	1.00	3.33	0.44	0.58	− 0.03
Psi-05	Venice	2500	65	15	15	11	0.73	2.30	0.89	2.06	0.53	0.36	− 0.31
Psi-06	Venice	20,000	65	20	20	20	1.00	3.00	1.00	3.61	0.52	0.63	− 0.60
Psi-07	Venice	1500	65	18	18	17	0.94	2.81	0.97	1.96	0.64	0.38	0.09
Psi-08	Venice	100,000	65	20	19	19	1.00	2.94	1.00	3.24	0.53	0.57	0.06
Psi-09	Venice	20,000	60	17	16	12	0.88	2.60	0.95	3.39	0.57	0.60	0.13
Psi-10	Venice	2000	60	17	17	17	1.00	2.83	1.00	2.62	0.42	0.49	0.26
Psi-11	Venice	3000	60	13	11	11	1.00	2.40	1.00	2.78	0.41	0.51	0.03
Psi-12	Venice	12,000	60	20	19	19	1.00	2.94	1.00	3.14	0.39	0.56	− 0.12
Psi-13	Venice	5000	60	20	18	11	0.61	2.14	0.74	2.04	0.35	0.34	0.16
Psi-14	Venice	500,000	60	20	18	18	1.00	2.89	1.00	3.13	0.42	0.53	− 0.24
Psi-15	Venice	60	50	20	18	16	0.89	2.74	0.95	2.93	0.61	0.56	− 0.02
Psi-16	Venice	20,000	50	16	15	15	1.00	2.71	1.00	3.05	0.42	0.52	− 0.48
Psi-17	Venice	500,000	60	20	19	15	0.79	2.52	0.72	2.54	0.60	0.48	0.18
Psi-18	Venice	5000	60	17	17	16	0.94	2.75	0.97	2.24	0.55	0.41	0.03
Psi-19	Venice	5000	60	14	14	14	1.00	2.64	1.00	2.57	0.53	0.51	− 0.13
Psi-20	Venice	5000	60	11	11	7	0.64	1.80	0.85	1.83	0.49	0.29	− 0.07
Psi-21	Genoa	20,000	95	20	20	18	0.90	2.86	0.96	3.11	0.49	0.54	0.25
Psi-22	Genoa	1000	95	20	18	13	0.72	2.45	0.86	2.42	0.55	0.47	− 0.25
Psi-23	Genoa	10,000	95	15	13	13	1.00	2.57	1.00	2.73	0.65	0.52	− 0.15
Psi-24	Genoa	150,000	95	20	18	9	0.50	2.01	0.84	1.67	0.46	0.32	− 0.73
Psi-25	Genoa	25,000	95	20	19	11	0.58	2.23	0.81	2.62	0.36	0.51	− 0.22
Psi-26	Genoa	15,000	95	20	18	17	0.94	2.81	0.97	2.88	0.40	0.49	0.12
Psi-27	Genoa	80	50	10	8	8	1.00	2.08	1.00	2.25	0.58	0.44	− 0.29
Psi-28	Venice	20,000	60	20	20	18	0.90	2.86	0.96	3.14	0.48	0.58	− 0.45
Psi-29	Venice	20,000	60	20	18	18	1.00	2.89	1.00	2.94	0.52	0.57	0.09
Psi-30	Venice	35,000	60	20	20	20	1.00	3.00	1.00	2.80	0.68	0.54	− 0.19
Psi-31	Venice	80	60	18	18	18	1.00	2.89	1.00	2.99	0.48	0.57	0.15
Psi-32	Bari	200	65	20	19	18	0.95	2.87	0.97	2.59	0.59	0.50	0.05
Psi-33	Bari	40	65	16	15	5	0.33	1.53	0.90	1.47	0.53	0.20	− 0.70
Psi-34	Bari	60	30	20	20	19	1.00	3.00	1.00	2.47	0.59	0.48	0.13
Psi-35	Venice	1000	65	13	12	12	1.00	2.49	1.00	3.31	0.38	0.56	− 0.19
Psi-36	Venice	1000	60	20	19	19	1.00	2.94	1.00	3.19	0.47	0.58	0.15
Psi-37	Venice	1000	65	20	20	15	0.75	2.60	0.87	1.67	0.32	0.22	− 0.24
Psi-38	Venice	1000	85	12	12	12	1.00	2.49	1.00	3.11	0.48	0.54	0.08
Psi-39	Venice	20,000	70	12	12	12	1.00	2.49	1.00	3.18	0.53	0.58	0.25
Psi-40	Venice	30,000	70	15	15	15	1.00	2.71	1.00	2.89	0.61	0.54	0.15
Psi-41	Venice	50,000	60	15	15	11	0.73	2.27	0.85	2.98	0.59	0.56	− 0.24
Psi-42	Venice	30,000	60	15	14	12	0.86	2.44	0.94	3.20	0.66	0.59	0.08
Psi-43	Montpellier	2000	70	18	15	14	0.93	2.62	0.97	2.29	0.31	0.42	0.03
Psi-44	Barcelona	30,000	60	15	13	13	1.00	2.57	1.00	2.13	0.39	0.46	− 0.63
Psi-45	Barcelona	20,000	60	17	17	10	0.59	2.04	0.70	1.63	0.60	0.27	− 0.70
Psi-46	Barcelona	15,000	50	17	17	11	0.65	2.12	0.70	1.88	0.61	0.33	− 0.56
Psi-47	Barcelona	20,000	60	14	13	10	0.92	2.46	0.96	2.34	0.56	0.41	− 0.13
Psi-48	Barcelona	30,000	45	16	16	16	1.00	2.77	1.00	2.13	0.61	0.40	− 0.28
Psi-49	Barcelona	100	35	16	16	8	0.50	1.96	0.89	1.85	0.41	0.36	− 0.79
Psi-50	N Europe	6000	120	20	19	16	0.84	2.70	0.89	1.83	0.59	0.36	− 0.57
Psi-51	N Europe	3000	70	14	14	13	0.93	2.54	0.96	1.61	0.34	0.24	− 0.32
Psi-52	N Europe	8000	120	13	13	6	0.46	1.63	0.82	1.63	0.41	0.23	− 0.68
Psi-53	N Europe	2000	120	16	16	9	0.75	2.39	0.89	1.66	0.42	0.25	− 0.53
Psi-54	N Europe	2000	110	14	14	12	0.86	2.44	0.94	1.72	0.40	0.26	− 0.39
Psi-55	N Europe	4000	105	17	16	8	0.50	1.81	0.73	1.43	0.28	0.18	− 0.36
Psi-56	N Europe	4000	115	17	16	10	0.63	2.10	0.76	1.76	0.43	0.27	− 0.48
Psi-57	N Europe	10,000	60	16	14	5	0.36	0.99	0.52	1.73	0.36	0.24	− 0.37
Psi-58	Bari	15,000	20	19	17	14	0.82	2.59	0.94	1.83	0.38	0.34	− 0.71
Psi-59	Bari	15,000	45	16	14	14	1.00	2.64	1.00	2.81	0.75	0.53	− 0.11
Psi-60	Bari	20,000	5	15	13	9	0.69	2.03	0.79	1.71	0.48	0.26	− 0.64
Psi-61	Bari	2000	15	18	18	12	0.67	2.32	0.82	1.55	0.54	0.22	− 0.32
Psi-62	Venice	10,000	60	20	19	14	0.74	2.52	0.87	1.91	0.59	0.37	0.09

Assuming that individuals belonging to the same MLG within a given population belong to the same genet, the ratio of the number of MLGs to the number of individuals genotyped in a population (G:N) gives an estimate of the degree of clonality within the populations (see Table 1). Mean G:N was 0.84 ± 0.19; the lowest value (0.33) was found in population Psi-33 (Foro di Ortona2), whereas Psi-34 (Marina Lesina) from the same region (Southern Adria, Bari) showed a ratio of 1.0 (Table 1). The highest mean G:N ratio at the regional level was observed in populations surrounding Montpellier (0.98 ± 0.04) and along the northern Adriatic coastline (= region Venice, 0.91 ± 0.12). The populations from Northern Europe were measured by an average G:N of 0.66 ± 0.21, indicating higher levels of clonality towards the north.

In 37 out of 60 populations we could identify identical MLGs within the population (Supplementary Table S2). Mostly, few ramets produced few clonal offspring, but some MLGs were represented by five and more sampled ramets. In case of Psi-57 (Bydgoszcz, Poland) one single MLG proliferated to even ten ramets sampled on an area of 1000 m^2^. Identical MLGs could be detected either in populations in the close neighborhood (Psi-52 and 53: Gerwisch 1 and 2, or Psi-46 and 47: Barcelona: Martorello and Montmelo) or in populations up to a geographical distance of 170 km (Psi-8 and 9: Eraclea Mare and Mali Lošinj, the latter indicating long-distance dispersal of vegetative propagules.

### Genetic diversity

Table 1 gives the details, and Supplementary Table S1 and Fig. S1 some additional statistics about basic regional differences of genetic diversity. The Shannon diversity index (H in Table 1) as a unifying measure of genetic diversity varies from 0.99 to 3.00. Evenness (E) as an alternative descriptor of genetic diversity is highly correlated with the Shannon index (Spearman’s r = 0.642; p < 0.001) and ranged from 0.52 to 1.0. Regional differences of H and E were significant in total (p = 0.005, and p = 0.011 resp.), but differences in H as well as E were significant only between N Europe (H = 2.08 ± 0.57, and E = 0.82 ± 0.14 resp.) and Venice (H = 2.64 ± 0.28 and E = 0.95 ± 0.08, resp.) (see Supplementary Table S1).

The mean allelic richness (Na) across all populations was 2.45 ± 0.62 when only unique MLGs were analyzed (cc, Table 1), and 2.65 ± 0.74 when all individuals were included (ori, Supplementary Table S2), being not significantly different (Mann–Whitney-*U*-Test: p = 0.350, see Supplementary Fig. S1). Na was not correlated (Pearson) with presumed population age, total population size or latitude, but proved to differ significantly by regions (Supplementary Fig. S1, all over Kruskal–Wallis-Test: p < 0.001), i.e. mean Na for the populations from Northern Europe was significantly (p < 0.001) lower than the mean for all other populations, regardless, if the calculations were either based on the original or the clone corrected data set.

The mean observed heterozygosity (*H*_*o*_, Table 1) ranged from 0.28 (Psi-55: Falkenberg in Sweden) to 0.75 (Psi-44: Platy el Prat, Barcelona). Pearson correlation coefficients showed that *H*_*o*_ was negatively correlated to latitude (R =  − 0.327, p = 0.011). ANOVA and consecutive Tukey-HSD-Test for significant group median differences revealed no effect of regions (p = 0.371), population age (p = 0.457), or population size (p = 0.069) on *H*_*o*_.

Average expected gene diversity (*H*_*e*_) across all populations was 0.43 ± 0.13 with the lowest within-population diversity (*H*_*e*_ = 0.18) observed in the Swedish population (Psi-55, Falkenberg) and the highest (*H*_*e*_ = 0.63) observed in population Psi-6 (Alberoni, Lido di Venice) (Table 1). Regional differences in *H*_*e*_ were significant in general (p < 0.001), and specifically differing when *H*_*e*_ median values of Northern Europe populations are compared with those from Montpellier (p = 0.026) and from Venice (p < 0.001). *H*_*e*_ was also negatively correlated to latitude in both data sets (cc: R =  − 0.365, p = 0.004; ori: R =  − 0.354, p = 0.006). There were no differences of *H*_*e*_ with respect to population size (p = 0.103), but differences by age classes (all over p = 0.049; Supplementary Table S1). However, specific age classes showed no significant pairwise differences.

The mean inbreeding coefficient (*F*_is_) was − 0.19 (± 0.30) (with negative *F*_is_ values appearing in 38 out of 60 populations (Table 1). When analyzing median *F*_is_ values with respect to regions significant differences (Kruskal–Wallis-Test: p < 0.001) were detected; populations of Venice differed significantly from those of Northern Europe (p = 0.004) as well as from those of Barcelona (p = 0.014). *F*_is_ showed no difference with respect to population size (p = 0.987). Age matters for *F*_is_ in general (Kruskal–Wallis-Test: all over p = 0.037); but the differences between the specific age classes were not significant with respect to median *F*_is_.

All populations showed significant deviations from Hardy–Weinberg equilibrium what indicates deficiencies of heterozygotes all over our sampled populations.

### Genetic structure

**Figure 1:**
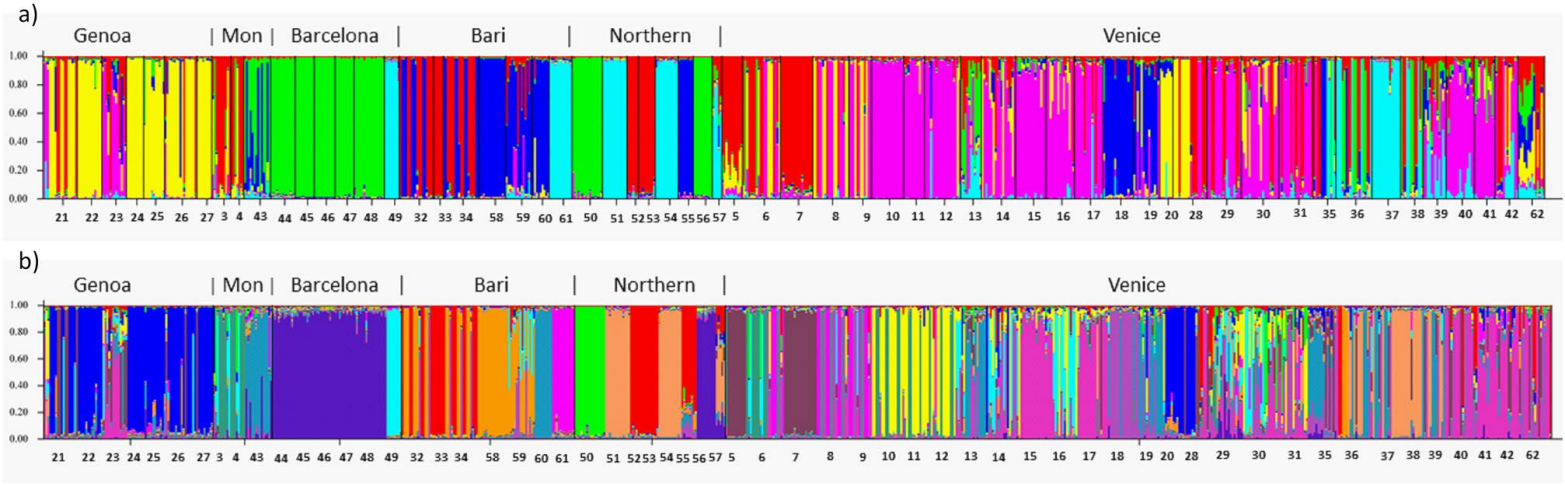
Assignment of 792 European *Ambrosia psilostachya* individuals to color coded genetic cluster levels *K* = 6 (**a**) and *K* = 14 (**b**) based on clone corrected SSR-data analyzed in STRUCTURE. Populations are arranged to six geographic regions (main harbor regions: Genoa, Montpellier, Barcelona, Bari, Northern Europe, Venice).

**Figure 2:**
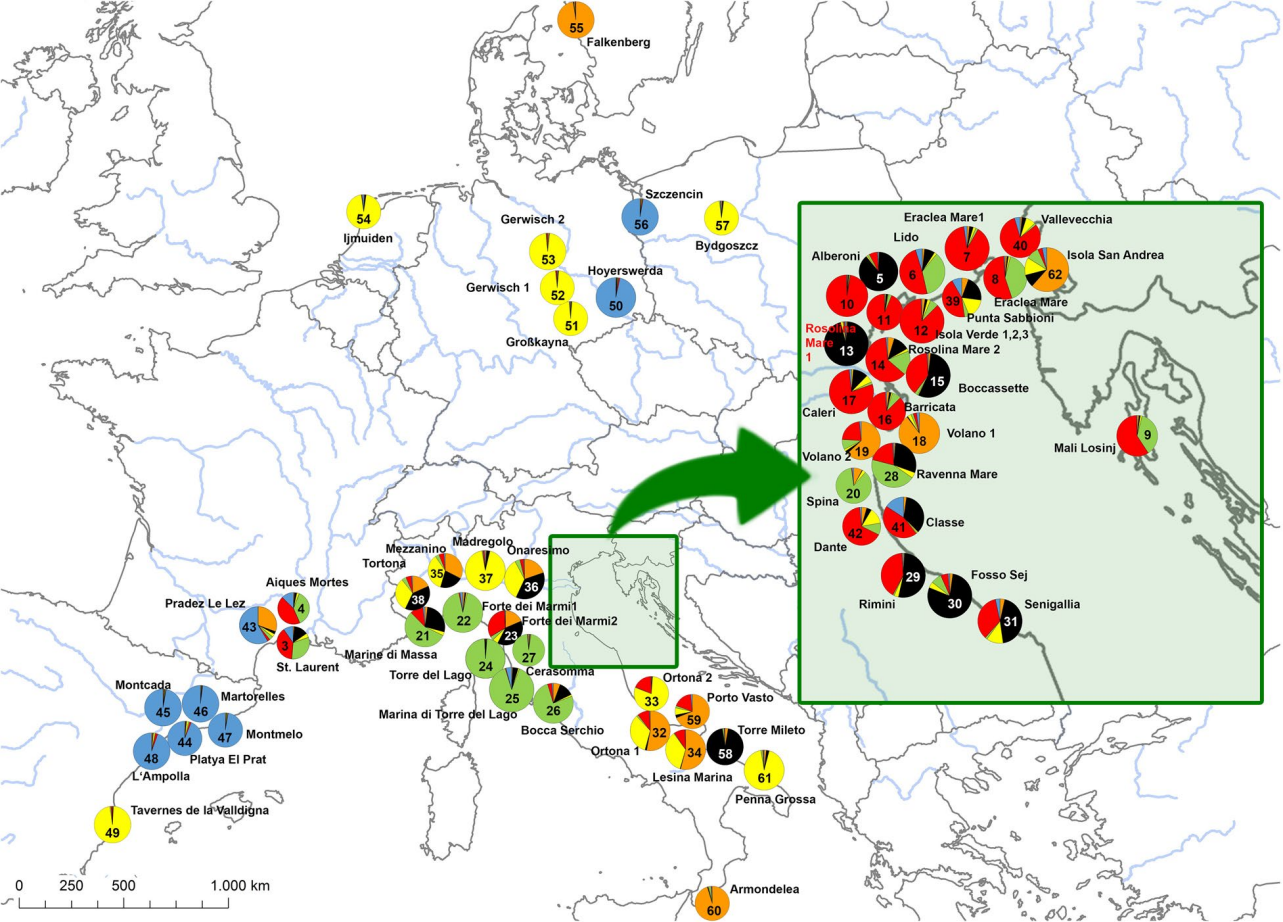
Map of sampled *Ambrosia psilostachya* populations in Europe. Pie charts show proportions of membership of individuals for the *K* = 6 groups of Bayesian Clustering (sizes of the pie charts refer to the population sample size; 8 to 20 individuals). The base map was generated using the free of charge software QGIS 3.22 (https://www.qgis.org/en/site/forusers/visualchangelog322/index.html); pie charts were created using MS Excel included in MS Office Professional Plus 2019.

Bayesian clustering analysis performed on either the original and the clone corrected data set revealed that populations could be assigned in both cases basically to six genetic groups (Fig. 1 & Supplementary Fig. S2). Both, the original as well as the clone corrected data set, revealed *K* = 6 and *K* = 14 as most likely number of groups what was supported by the log likelihood lnP(D) calculations (Supplementary Fig. S3). Interestingly, the six genetic groups correspond largely with geographical locations, which could be partly affiliated to predefined regions (Figs. 1, 2, column “region” in Table 1). The centres of these regions were characterized as either important European trading harbors in Southern Europe (i.e. Barcelona, Bari, Genoa, Montpellier, and Venice) probably functioning as primary starting points of introductions, or the whole non-Mediterranean “Northern Europe” region (including Central Europe). The populations in Northern Europe (corresponding largely to the yellow genetic group in Fig. 2) are scattered over four different countries and could not be affiliated to any specific trading port. However, three out of eight northern populations (Psi-50, Hoyerswerda in Germany and Psi-56, Szczecin in Poland, both marked blue; and Psi-55, Falkenberg in Sweden, marked orange;) showed a completely different genetic constitution than the rest of the Northern European populations. They obviously share genetic variation mainly distributed in the Mediterranean regions of Barcelona and Bari, respectively.

Generally, the degree of admixture at the population level differs fairly between regions and clusters. The populations in the North and in Spain are commonly most homogenous, i.e. dominated by individuals affiliated to a single genetic group. Most populations along the coastline of Tuscany (east of Genoa) also showed relatively low levels of admixture. The populations around Montpellier, in the Venice region (including the Po valley), and along the Southern Adriatic coastline (Bari region) are characterized by high degrees of admixture on average. Given these high levels of admixture in Bayesian Clustering, Principal Coordinate Analysis not surprisingly indicated relatively weak genetic structure (Supplementary Fig. S4) with the first two axes explaining only 8.6%, and 6.4% resp., of the total genetic variation.

Mantel tests imply significant isolation by distance (Supplementary Fig. S5). Spatial autocorrelation analysis revealed significant geographic correlation among all 60 populations (p = 0.001) as well as among the populations within regions (each with p = 0.001). Spatial autocorrelation test of the whole data set (Supplementary Fig. S6) revealed that the smallest distance classes (± 200 km) showed a clear positive relatedness of the individuals (r-values higher than expected, going far beyond the 95% confidence level) whereas at the intermediate geographical distances the relationship was rather lower than expected. However, towards one of the highest distance classes (about 1800 km) the relationship got positive again. The spatial autocorrelation analysis within the single regions (Supplementary Fig. S7) gave again positive relatedness of the individuals at low geographical distances within the respective regions.

### Genetic differentiation among geographic regions

Hierarchical AMOVA on the full data set and all 955 individuals (ori data set) affiliated to the predefined regions showed that 10.4% of genetic variation occurred among these regions, and 40.3% of the genetic variation was found among populations within regions (Table 2). Most genetic variation was observed among individuals within populations (49.2%). The AMOVA performed on unique MLGs (cc data set) showed similar results: 9.9% of the genetic variation was found among regions, 34.0% among populations and 56.1% among individuals. Particularly within populations that are geographically associated to the surrounding of Barcelona as well as within the populations from Northern Europe (Netherlands, Germany and Poland) genetic variation among populations was significantly higher than among individuals (Table 2).

**Table 2:** Hierarchical analysis of molecular variance (AMOVA) of *Ambrosia psilostachya* based on 999 permutations for the whole dataset and for the defined European regions, separately. Values without parentheses indicate estimates with the original data set, values in parentheses estimates based on the clone corrected data.

Source of variation	Degrees of freedom	Variance estimated	% of variance
All regions			
Among regions	5 (5)	0.6 (0.6)	10.4 (9.9)
Among populations	54 (54)	2.4 (2.0)	40.4 (34.0)
Among individuals	895 (732)	2.9 (3.4)	49.2 (56.1)
Within Barcelona			
Among populations	5 (5)	3.0 (2.7)	72.4 (65.1)
Among individuals	86 (64)	1.1 (1.4)	27.6 (34.9)
Within Bari			
Among populations	6 (6)	3.3 (2.7)	58.9 (49.5)
Among individuals	109 (85)	2.3 (2.8)	41.1 (50.5)
Within Genoa			
Among populations	6 (6)	1.5 (1.3)	32.8 (26.4)
Among individuals	107 (82)	3.2 (3.6)	67.2 (73.6)
Within Montpellier			
Among populations	2 (2)	1.6 (1.6)	26.8 (26.0)
Among individuals	29 (28)	4.4 (4.5)	73.2 (74.0)
Within Northern Europe			
Among populations	7 (7)	5.1 (5.0)	87.9 (84.6)
Among individuals	114 (74)	0.7 (0.9)	12.1 (15.4)
Within Venice			
Among populations	28 (28)	1.7 (1.5)	30.9 (27.0)
Among individuals	450 (407)	3.8 (4.1)	69.1 (73.0)

## Discussion

### Clonality remarkably affects the population genetics of introduced *A*. *psilostachya*

We identified substantial clonal reproduction within populations of *A. psilostachya*, but also dispersal of clonal offspring between populations throughout Europe. Duplicated MLGs were found in 62% of the analyzed populations. This can be expected from the morphological traits of creeping roots and added buds producing shoot sprouts from belowground^34^. We provide prove for this obvious species trait based on genetic data for the first time. Expecting an important role of clonal reproduction in *A. psilostachya* our sampling strategy was to collect individual ramets at a minimum distance of one meter. Due to our sampling strategy, we expected that our sampling might catch enough ramets from different genets to perform a reasonable analysis of genetic variation. Wagner & Beals^24^ stated, from their explorative analysis of *A. psilostachya* in the field, that clonal exploration of genets might cover many meters distance. We also observed secondary thickening of the horizontal roots as well as expansion of still connected roots of three meters length. This allows the assumption of a relatively long-lived clonal integration.

Besides clonal propagation, sexual reproduction plays a role in population dynamics of *A. psilostachya*^34^. The degree of sexual reproduction can vary from one individual clone to another by many to no fruits produced^31^. Furthermore, the latter authors stated that individual clones may differ also by specific, but in their study not detected, morphological characters. In general, identification of different genets can be indicated from MLG heterogeneity at the population level. Interestingly, our analysis provided evidences in various directions. Based on Wagner & Beals^24^ clonal growth can expand up to hundreds of m^2^ in *A. psilostachya*. Consequently, we expected to find clonal duplicates, especially in older spatially structured populations that had enough time to expand by hectares. Interestingly, this phenomenon could be found only in one case: Psi-51 and Psi-52 (two locations about 200 m apart, in a former military training area in Germany), but not in the case of subpopulations along the coast near Venice (Isola Verde 1, 2, 3; Rosolina Mare 1, 2; Lido di Volano 1, 2) – each population at only about 100–200 m apart from its neighbor. Lack of human disturbance due to conservancy regulations may have limited these small parceled coastal populations in the Venice region to spread one hundred or more meters or more. In contrast, the military training area in Germany was formerly utilized by tanks that can easily transport contaminated sandy soil containing ragweed root fragments^60^ over hundreds of meters. Dispersal of either seeds or clonal fragments of *A. psilostachya* over long distances of up to 170 km from and to coastal dunes was also proved by our data (Supplementary Table S2). As carrying vector, we are considering contaminated vehicles used for construction of infrastructure along beaches. Successful transportation of seeds by vehicles is well documented, at least for *A. artemisiifolia* in Europe^61–63^.

Another likely mode of dispersal is transportation by Adriatic Sea currents: the main direction of sea current along the Italian Adriatic coastline from North to South^64^ could have transported plant fragments. This would fit to the fact that the first occurrences of *A. psilostachya* were documented in areas surrounding Venice in the late thirties of the twentieth century whereas the Bari region was occupied decades later (Supplementary Table S3). Unfortunately, nothing is known about the survival of seeds or root and shoot fragments of our study species in sea water. However, Fumanal et al.^65^ showed that seeds of the related taxon *A. artemisiifolia* partially succeeded drifting in water and were still germinable after several days of inundation. Dispersal of clonal fragments of the invasive *Carpobrotus edulis* in floating sea water by sea currents up to 250 km within a few days was modelled comprehensibly based on the high survival rates of its clonal fragments in sea water^66^. Consequently, additional physiological experiments might enlighten the potential for transportation of plant fragments by sea current in case of *A. psilostachya*. However, our genetic data are not in conflict with this potential dispersal vector.

### Genetic diversity varies along spatial and/or temporal trajectories

Parameters on genetic diversity can elucidate processes and directions of invasions. Invasion processes of plants with at least partial clonal reproduction tend to conserve diversity phenomena of initial colonization and subsequent local dispersal^67^ due to successful reproduction without seeds. Even local spread by ramet fragments can be indicated from such genetic data, since we proved local spread of unique MLGs over distances of up to 170 km (Supplementary Table S2).

Population genetic parameters may differ when MLG duplicates are either involved or removed from the data set^53,67,68^. In contrast, we found no significant differences at the population level when comparing allelic richness, *H*_*o*_, *H*_*e*_, and *F*_is_ for the whole data set calculated either for the original or the clone corrected data. However, all these parameters except for observed heterozygosity (ori: p = 0.090, cc: p = 0.069) differed significantly by regions (Supplementary Fig. S1a, Kruskal–Wallis-Test, p = 0.005) and partially − only for young populations − by age of the population, but not by population size (p = 0.971). Interestingly, mean observed heterozygosity *H*_*o*_ was marginally not significantly lower in Northern Europe populations than in all Mediterranean populations. Therefore, arrival and long-lasting persistence of genets from different source populations seems to be typical for *A. psilostachya*, but repeated/multiple introductions happened more frequently towards the south.

Our values for observed heterozygosity (mean *H*_*o*_: 0.41 ± 0.09) of the perennial *A. psilostachya* correspond to *H*_*o*_-estimate levels of the annual *A. artemisiifolia* (0.21–0.76) given by various authors (i.e. ^38,44,45,49,69^). Van Boheemen et al.^46^ gained on average lower *H*_*o*_-values (0.16–0.30) for *A. artemisiifolia*, likely due to the use of SNPs (not SSRs) and to admixture processes. Mean values of expected heterozygosity of *A. psilostachya* were similar for the original and the clone corrected data. This corresponds to invasive *Carpobrotus acinaciformis* showing also no effect of clone correction in this respect^70^. Differences of *H*_*o*_ and *H*_*e*_ in *A. psilostachya* were significant when comparing the populations of Northern Europe, Bari and Barcelona regions (Mann–Whitney-*U*-Test: p < 0.001). Thereby, Northern Europe and Barcelona cover populations with low levels of admixture (Fig. 2).

### High levels of heterozygosity excess in small populations

We found strikingly low values of *F*_is_, far below zero for both the original as well as the clone corrected data in most regions except for Venice and Montpellier. Such negative values indicate excess of heterozygosity. Stoeckel et al.^71^ demonstrated some slight but not significant difference of original and clone corrected *F*_is_ -values for *Prunus avium* in France. Even more strikingly negative *F*_is_ population means were found in *Carpobrotus acinaciformis* that reproduces indeed almost exclusively by clonal fragmentation: Suehs et al.^70^ found very strong heterozygous excess (− 0.659, on average) for the original data set (calculated for all sampled ramets) and still significantly negative values (− 0.371, on average) for clone corrected data. In comparison, the related *C. edulis* showed rather homozygosity excess possibly due to partial selfing.

The strictly negative *F*_is_ -values of many *A. psilostachya* populations are in contrast to those of the annual *A. artemisiifolia* that turned out to have significantly positive deviation from Hardy–Weinberg equilibrium in practically all publications available^38–40,43,49^. *A. psilostachya* populations in Europe are exposed to various effects that may cause distinct excess of heterozygosity. In case of small absolute population sizes, Balloux^72^ stated that there is a clear tendency to heterozygosity excess due to small effective population size (i.e. few sexually reproducing individuals). Such could hold for our small and old populations (relicts) in Northern Europe, where we have to assume problems with seed production and the dominancy of few well-adapted clones. But very small-sized Mediterranean populations with high genetic variation possibly represent remnants of formerly bigger populations that eroded along the sandy coastline (i.e. Psi-15, Psi-31, and Psi-34). The longevity of successful, but few genets in isolated populations can go along preference of favoring basically heterozygous individuals over homozygous due to heterosis^73^ and inducing an excess of heterozygosity also in neutral loci of the genome by hitch-hiking^71^.

Balanced *F*_is_ values were found in larger populations. However, in this case low sexual reproduction rates and spatially restricted dispersal may cause sub-structuring of even large populations^71^. Such could have happened with three nearby populations Psi-10, -11, and -12 (Isola Verde, dunes south of Venice, subpopulations only 40 and 100 m apart) that possibly developed from one founder population a long time ago, because they show ± the same degree of admixture (Fig. 2), but interestingly no clonal duplicate in common (Supplementary Table S2); on the other hand, our sampling could not detect any clonality within the single samples of these very nearby populations. Nevertheless, we found significant linkage disequilibria among loci, populations, and regions, which is – together with negative *F*_is_—a main characteristic of asexual or partially asexual (clonal) populations^57,68^. As self-incompatible species produce no offspring through selfing, this can lead to a significant deviation from random mating (i.e. in small populations), significantly influencing *F*_is_ and generating heterozygote excess^72^. This factor might have caused genetic erosion of some few older populations of *A. psilostachya*. Distinct negative *F*_is_ means at population level may also be indicative of an insufficient number of MLGs represented in the samples^74^.

### Population genetics reflect the age of small populations (old relicts versus young founders)

There is no clear mono-factorial picture in our data. In a very isolated small population with very few ramets (Psi-34, Lesina Marina, Bari region) all 20 sampled ramets (out of a total of 60 ramets) turned out to represent unique MLGs (G/N = 1, *F*_is_ = 0.13). Comparable situations were observed in Psi-31 (Senigallia, Venice region) and Psi-15 (Boccasette, Venice) with population totals of 80 and 60 ramets, and 18 sampled ramets each that were almost all unique MLGs (G/N = 1, and 0.89 resp.; *F*_is_ = 0.15, and 0.03 resp.). In all three cases the sites suffered from coastal erosion at the front line of old grey dunes. This argues for historical (old) admixture at place. Other small populations like Psi-27 (Cerasomma, Genoa region, 15 out of 40 ramets sampled), Psi-33 (Foro di Ortona2, Bari, 8/80 ramets) and Psi-49 (Tavernes de Valldigna, Barcelona, 16/100 ramets) represent spatially delimitable populations of only few m^2^ each, at young roadsides that were obviously of recent age. While Psi-27 was represented only by unique MLGs (G/N = 1), the other two populations comprised of several multiplied genets (G/N = 0.33, and 0.50 resp.). In consequence, the mean *F*_is_ of Psi-27 was moderate (−  0.29) whereas the other two populations showed strongly negative values of *F*_is_ =  −  0.70 and −  0.79, respectively. These very low population means of *F*_is_ might indicate significant undersampling^74^ or an initial population undergoing a bottleneck phase^67,72^, respectively. Based on our data we could not identify any bottleneck effects, in general. Small but older populations like Psi-15, Psi-31 and Psi-34 obviously consisted of several MLGs that may have survived since foundation due to clonal persistence and may therefore still represent the genetic structure of the initial colonization stage.

### Population genetics comparison between the perennial and the annual invader

Most population genetic studies on the annual *A. artemisiifolia* were performed at the continental^35,38,39,42,45,46,48,49^ or regional^40,41,43,44^ scale using different SSRs, AFLPs or SNPs. These macro-scale analyses, as well as the local analysis by Kropf et al.^47^, based on AFLPs, demonstrated high admixture rates for nearby populations due to probable high levels of gene flow and repeated introductions from multiple sources. Only Kočiš Tubić et al.^43^, based on SSRs, found some decrease of genetic differentiation between populations in Serbia that might be correlated to geographical distance. The macro-scale analyses of *A. artemisiifolia* population genetic studies cited above deduced repeated introductions including several genetic consequences^75,76^ and secondary spread at high rates for the invasive range of the annual *A. artemisiifolia* across Europe, resulting in the lack of isolation by distance. However, we found that perennial *A. psilostachya* also represents an invasive species that shows no clear isolation by distance considering all populations and therefore, also a highly dynamic, ongoing invasion history, especially in Southern Europe. Isolation by distance was found in some regions only; although the respective R-values were low (Supplementary Fig. S5).

In case of *A. psilostachya*, we found relatively high degrees of admixture in some Mediterranean regions but not for the North European and Spanish populations (Figs. 1 and 2). The degree of admixture is specifically high in the Venice region (see inset of Fig. 2) where almost all sandy beaches are populated by *A. psilostachya*. While *A. artemisiifolia* is well known as agricultural weed transported throughout Europe in crop seed containments^12,77,78^, therefore, resulting in high degrees of admixture all over Europe, *A. psilostachya*, in contrast, rarely occurs on crop fields^3^, but in more natural habitat types. Therefore, and due to its main vegetative reproduction mode, current genetic diversity of populations represents rather the diversity of the founder population. This is most plausible for North European introductions that have survived for decades clonally. In case of Southern Europe, the older populations might have added slightly differing genotypes generated from locally produced seeds. At least in populations from southern France, Fried et al.^3^ showed that *A. psilostachya* is able to produce viable seeds at rates of 10 − 20%.

In general, founder populations of species expanding their range undergo selection of genotypes resistant to environmental constraints. Additionally, surviving clones do not need to reproduce by seeds due to bottleneck situations and unfavorable environmental stressors. Northern European initial populations of *A. psilostachya* might have faced this problem, indicated by their low Na and *H*_*e*_, but also by highly significant negative *F*_is_-values. To form extant populations via vegetative expansion takes time and our invasive study species expands in space mostly vegetatively. This is why all populations that comprise ≥ 50,000 individual ramets are at least at an age of 60 years or older. Genetic differentiation among populations within a region is lowest for Venice region where there is a high density of populations or subpopulations. If populations are geographically closer in this region, more dispersal events of single clonal fragments among them is to be expected. This is indeed the case for several adjacent populations around Venice (Supplementary Table S3).

## Conclusions

Parameters describing population genetics of the invasive *A. psilostachya* did not differ with respect to calculations based on all sampled ramets or on unique genets only. This indicates that the sampling was sufficient for respective estimates. The strikingly negative inbreeding coefficient of small populations indicates the long-term persistence of well-adapted heterozygous clones. Under harsh environmental conditions, survival and expansion at the population level by clonal offspring can, therefore, be advantageous against the establishment by seeds^79,80^. Establishment and, specifically, spatial expansion can be less risky if root sprouts are produced that overcome stressful periods by resource allocations from the connected belowground mother individual organs^81^. Seedlings are commonly less costly but they easily face severe problems along with germination and juvenile growth if the environment is too harsh. In case of *A. psilostachya,* the vegetation period in Northern Europe is simply too short to produce ripened seeds, but long enough to assimilate and store carbohydrates belowground for renewal next season. In consequence, population genetics of the clonal *A. psilostachya* resembles the historical constitution of the founder populations. Change rates of population genetic parameters during the invasion process may be rather low, either with respect to expansion or reduction of clonal patches. Local extinction of such clonal species may take far longer than in short lived outcrossing annuals^79^. If genets get very old, they can accumulate somatic mutations, pathogens and epigenetic traits that may also contribute to genetic variation, hitchhiking even in neutral markers^81^. A future comparative detailed study of big and small, as well as, old and young populations would help to elucidate such processes of clonal population dynamics using population genetic parameters.

We learned that in clonal invaders, like western ragweed, even small populations may have a conservative history in that successfully established clones persist long time at the same place. Furthermore, bottleneck effects may be overcome easier than in outcrossing populations. We found a clear difference in naturalization dynamics and secondary spread throughout Europe. The perennial *A. psilostachya* forms long-lived local populations in Northern Europe but, recently, also larger populations towards the south. It performs far better with respect to recent spreading either by root fragments or seeds in the Mediterranean. But western ragweed is bound to instable habitat types everywhere, like river beds and coastal dunes. Prospectively, the invasion history in Europe might become clearer after consideration of North American source populations of western ragweed.

## Methods

### Study species and population sampling

In 2015 and 2016 a group of specialists evaluated several herbarium specimens of *A. psilostachya* from European herbaria^11^. Based on this experience we draw a map of ragweed occurrences in Europe to select for representative populations stretching the whole European invasive distribution range. Karrer et al.^29^ and Montagnani et al.^21^ report that some occurrence data from literature were due to misidentifications, named “*A. maritima*” in the south (Italy, Spain) or “*A. artemisiifolia*” in the north, respectively. Our sampling of verified *A. psilostachya* finally resulted in 60 populations listed in Table 1 & Supplementary Table S4. *A. coronopifolia* Torr. & A. Gray that was and is still in use in some databases^82,83^ and older literature is synonym to our taxon. Ten of the selected populations were tested for nuclear DNA-contents providing graphs that indicate identical ploidy levels relative to the respective reference (Supplementary Table S5).

Herbarium and literature studies revealed data on the first documented introductions to different European countries (Supplementary Table S3). The population size of sampled populations was estimated roughly in the field based on the number of visible shoots (Table 1). Furthermore, the prospective age of the populations was estimated based on the data about first introductions to the ‘regions’ in the surrounding of important European harbors (Table 1 and Supplementary Table S3). All ‘regions’ were geographically distinct except for ‘Northern Europe’ that includes all introductions into temperate Europe. Italy was invaded at different times; along the Tyrrhenian and North Adriatic coast line in the first half of the twentieth century, but along the South Adriatic coast line in the second half of the twentieth century or even later. Consequently, the Italian populations were affiliated to three different regions (Supplementary Table S4).

In each population we took maximum three leaves per individual stem and dried them quickly using silica gel. Commonly, we sampled leaves from maximum 20 individual stems per population at a distance of at least one meter from each other, except for very small populations that comprised only from few stems mostly nearby. In total, leaves from 1005 individuals were sampled from eight European countries. Furthermore, we collected few complete specimens for documentation. Specimens from all populations were identified by the first author and deposited in the herbarium collection of the University of Natural Resources and Life Sciences Vienna (WHB, cf. Supplementary Table S4). We confirm that the authors sampled and handled the collected plant material in accordance with the relevant institutional, national, and international guidelines and legislation. Our species of interest is alien to Europe and therefore we naturally comply with the IUCN Policy Statement on Research Involving Species at Risk of Extinction and the Convention on the Trade in Endangered Species of Wild Fauna and Flora.

### SSR genotyping and statistical analysis

For DNA extractions, one small fragment of each leaf sample was placed in a 2 ml microtube containing one steel bead and 400 µl extraction buffer^84^. Microtubes were agitated in a bead mill homogenizer, then incubated in a water bath 5 min at 95 °C, cooled on ice and centrifugated 2 min at 20,000 g. The resulting DNA extracts were stored at − 20 °C. Genotyping was performed at GENTYANE (INRAE, Clermont-Ferrand, France) based on 15 microsatellite loci (AMBELssr-EST71, -ill75, -ill101, -EST114, -EST150, -EST13, -ill64, -EST69, -ill55, -ill35, -ill02, -EST111, -ill18, -ill20, -EST54) previously developed at INRAE Dijon, France for *A. artemisiifolia*^45^. PCR products were labelled with one of four fluorescent tags (i.e. 6-FAM, NED, VIC or PET) and loaded on an ABI 3730XL capillary DNA analyzer (Applied Biosystems) using the size standard GS500 LIZ. We used Peakscanner version 1.0 (Applied Biosystems) to read allele sizes. Poorly performing individuals with missing genotype call were excluded from further analysis to avoid bias in the data analysis^58^.

The presence and frequency of null alleles was checked for each marker using R-package “PopGenReport” version 3.0.4^85^ estimating a bootstrap confidence interval for each locus using the methods of Brookfield^86^.

For identification of unique multilocus genotypes (MLGs) and genotypic (clonal) diversity of *A. psilostachya* the minimum genetic distance was calculated from a relative dissimilarity distance matrix (threshold = 0.5^58^). The number of 15 loci used in our study fits to the needs for detecting unique MLGs^59^. Using these identified MLGs per population (“original” [ori] data set), we estimated the following components of genotypic diversity in each population: Shannon’s diversity index (H), genotypic evenness (E), and the ratio of number of MLGs to the number of individuals analyzed (genets to ramets = G:N). G:N indicates the degree of clonality within the population. A value of one indicates purely sexual reproduction, while a value near zero indicates purely clonal reproduction.

In clonal organisms such as *A. psilostachya*, asexual reproduction can result in a single genet being represented by multiple physiologically independent ramets^87^. Thus, we identified shared multilocus genotypes (MLGs) within or (even) across different populations using R-package “poppr” version 2.8.2^58^, which can be used to infer clonal membership to the same genet^87^. We kept only one of each MLG per population in the “clone corrected” (cc) data set that served finally for additional calculations of the genetic diversity measures H, E and allelic richness.

As the concept of a population representing a group of interbreeding individuals is not straight forwardly applicable to species with clonal as well as sexual reproduction significant deviations from Hardy–Weinberg equilibrium (HWE) can be expected, as sexual and asexual reproduction may occur in parallel^88^. To determine deviations from HWE in the 60 European populations of *A. psilostachya* GenAlEx version 6.502^89^ was used. Tests for conformity with HWE were based on Chi-square tests, determining whether the observed genotypic frequencies are deviating from expected frequencies by chance or due to a lack of random mating.

Furthermore, we checked for linkage disequilibrium (LD) based on 999 permutations using R-package “poppr” version 2.8.2 testing if populations are clonal (where significant disequilibrium is expected due to linkage among loci) or sexual (where linkage among loci is not expected), expressed by the index of association (I_A_) proposed by Brown et al.^90^. Fixation index (*F*_ST_) matrices based on pairwise *F*_ST_ values were calculated for all pairs of populations and regions using R-package “hierfstat” version 0.04–22^91^.

In addition, population structure was assessed using STRUCTURE 2.3.4^92^. The admixture model and correlated allele frequencies between populations were selected as specified by Falush et al.^55^ to determine the number of genetic clusters (*K*) best fitting the data^93^. The length of the burn-in period was 100,000 runs followed by 500,000 Markov Chain Monte Carlo permutations. 20 iterations were performed for each value of *K* ranging from 1 to 15. Optimal values of *K* were determined based on log likelihood values as described by Pritchard et al.^92^. Principal Coordinate Analysis was calculated to show the interference of all individuals based on their genotypic similarity using Nei’s genetic distances^94,95^.

In the next step, spatial autocorrelation analysis was performed using GenAlEx version 6.503^89^ to describe the degree of similarity among regions and among populations within the regions. In addition, isolation by distance (IBD) was analyzed using R-packages “adegenet” version 2.1.1^96^ and “MASS”^97^ based on Mantel test with 999 replicates between a matrix of Euclidean distances and a matrix of geographic distances. Spatial autocorrelation test in GenAlEx was based on the distribution of permuted (r_p_) values under the assumption of no spatial structure and 999 random shuffling of individuals among distance classes.

All genetic diversity calculations including null allele frequency analysis, HWE, *I*_A_, and *F*_ST_-matrices were performed for two data sets, one containing all individuals, including multiples (duplicates) of the same MLGs (“ori”), and a second data set including only unique MLGs, subsequently called “clone corrected” data set (“cc”). Allelic richness per population was estimated using a rarefaction approach to correct for differences in populational sample size using R-package “diveRsity” version 1.9.90^98^. In addition, we estimated observed heterozygosity (*H*_*o*_), expected heterozygosity (*H*_*e*_), and the inbreeding coefficient (*F*_is_) for both, the ori and the cc data.

Confidence intervals for *F*_is_ were obtained using 999 bootstrap permutations. Moreover, a hierarchical Analysis of Molecular Variance (AMOVA^99^) for populations and regions was performed using the R-package “adegenet” version 2.1.1^96^ with statistical significance testing based on 999 permutations.

To analyze the genetic structure among different groupings, each genetic parameter was at first tested by Kolmogorov–Smirnov for normality. In consequence, *Ho* (ori and cc) and *F*_is_ (ori and cc) were treated by ANOVA and consecutive Tukey HSD for testing significant group median differences, and all other parameters were tested by non-parametric ranked Kruskal–Wallis-Test followed by a post-hoc Mann–Whitney-*U*-Test for significant group median differences. We tested for differences by six regions, four population age classes and five population size classes applying a significance level of p < 0.05 after adjusting by the Bonferroni correction for multiple tests (cf. Table 1). Population age was estimated from documented first occurrences until 2019 in the respective neighborhood of the 60 populations (see Supplementary Table S3) and classified into age class 1 (5–20 years), 2 (21–60), 3 (61–99), and 4 (≥ 100). Population size was classified into 1 (40–99 ramets), 2 (100–999), 3 (1,000–9,999), 4 (10,000–99,999), and 5 (100,000–500,000).

## Supplementary Information

Supplementary Information.

## Data Availability

Source data on the distribution of *Ambrosia psilostachya* in Europe and the full *F*_ST_-matrix can be provided on a reasonable request by the first author. All other data generated or analyzed for this study are included in this published article.
