# Supplementary material for: Genetic structuring and invasion status of the perennial Ambrosia psilostachya (Asteraceae) in Europe
Source: Sci Rep. 2023 Mar 6;13:3736. doi: 10.1038/s41598-023-30377-6 (PMC9988885; doi:10.1038/s41598-023-30377-6)
Supplement: Supplementary file 1 — Supplementary Information. [file 41598_2023_30377_MOESM1_ESM.pdf]

Supplementary material

Genetic structuring and invasion status of the perennial *Ambrosia psilostachya (Asteraceae)* in Europe

Gerhard Karrer, Rea Maria Hall, Valerie Le Corre, Matthias Kropf

Supplementary Figure S1: Overview of **a**) allelic richness (Na), **b**) observed heterozygosity (H_o), **c**) expected heterozygosity (H_e), and **d**) the inbreeding coefficient (F_{is}) of *Ambrosia psilostachya* populations associated to six European regions; the red line in d) marks the zero-line for the F_{is} coefficent; results below this line indicate inbreeding in populations/regions; analysis was performed on the original data set (955 individuals; light grey boxes) as well as the clone corrected data set (807 genets; dark grey boxes).

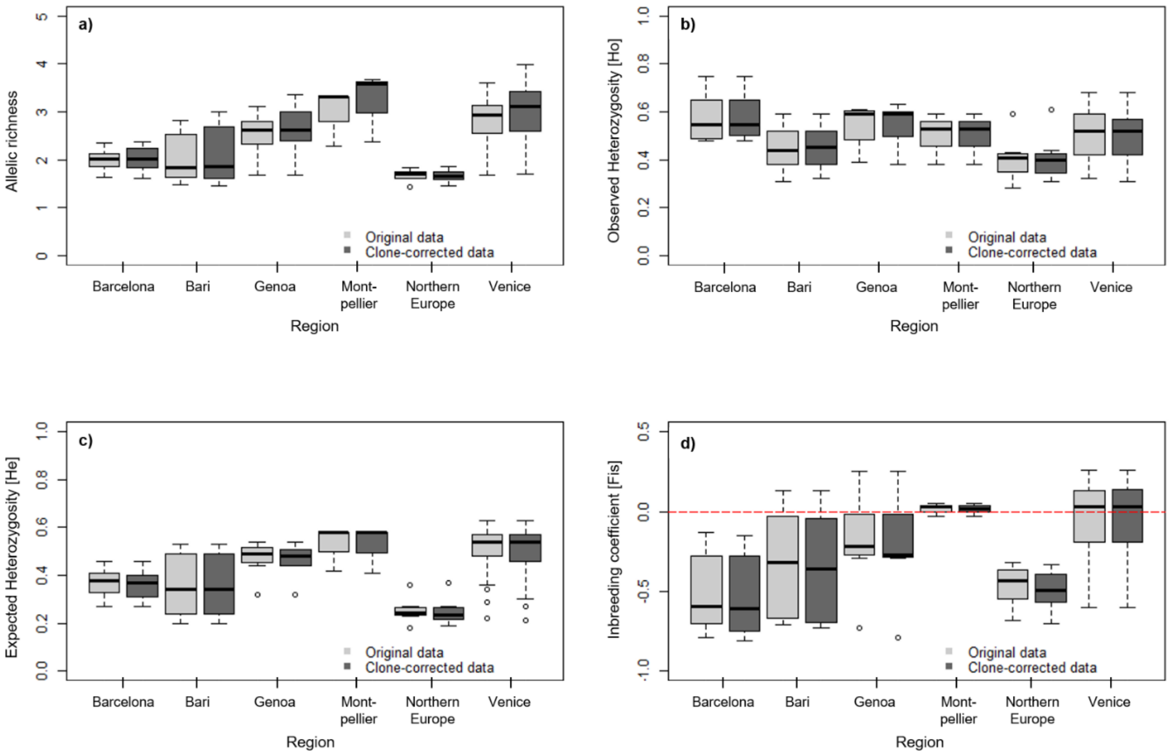

Supplementary Figure S2: Assignment of 955 European *Ambrosia psilostachya* individuals to color coded genetic cluster levels *K*=6 (**a**) and *K*=14 (**b**) based the original SSR-data analyzed in STRUCTURE. Populations are arranged to 6 geographic regions (main harbors).

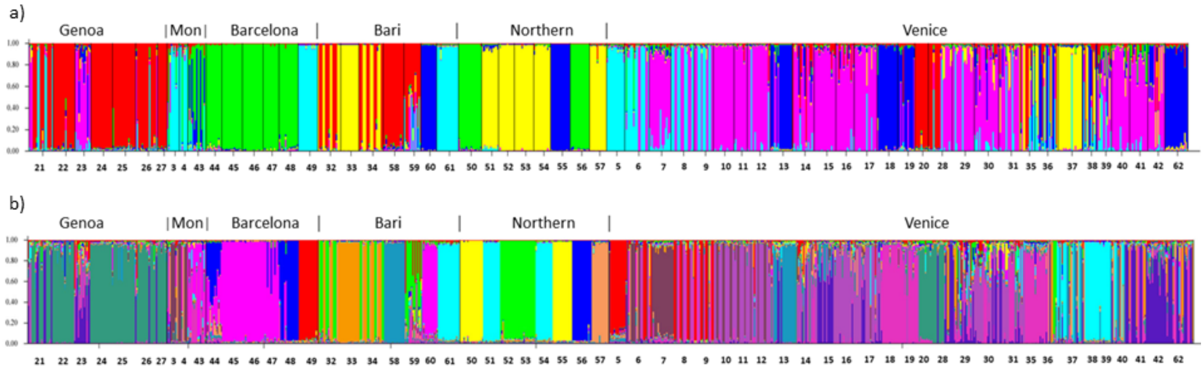

Supplementary Figure S3: Mean log likelihood lnP(D) as a function of genetic *K* clusters, based on 955 individuals of *Ambrosia psilostachya* from across Europe; calculated in Bayesian Clustering for the original and the clone corrected data; lnP(D) is based on twenty runs at each *K*-level.

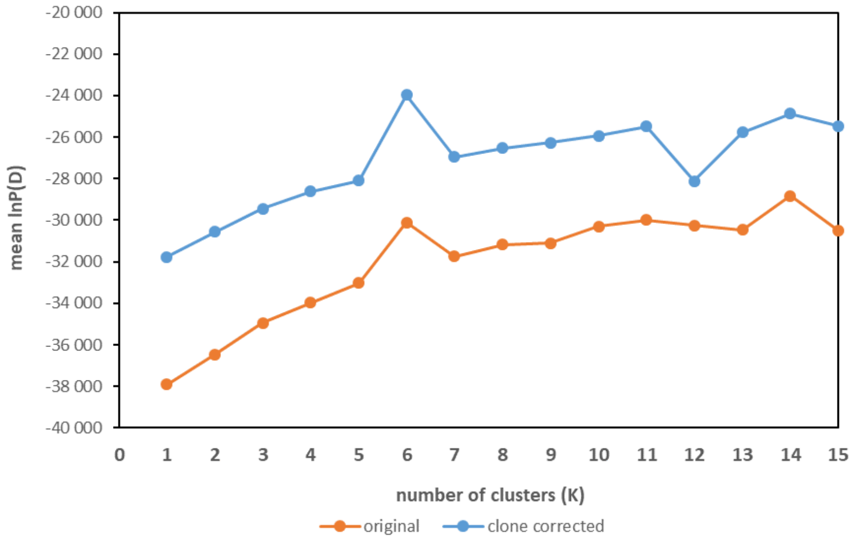

Supplementary Figure S4: Principal coordinate analysis of 792 *Ambrosia psilostachya* individuals from 60 European populations based on Nei's genetic distance of individual SSR genotypes; individuals are affiliated by different colors to the predefined regions.

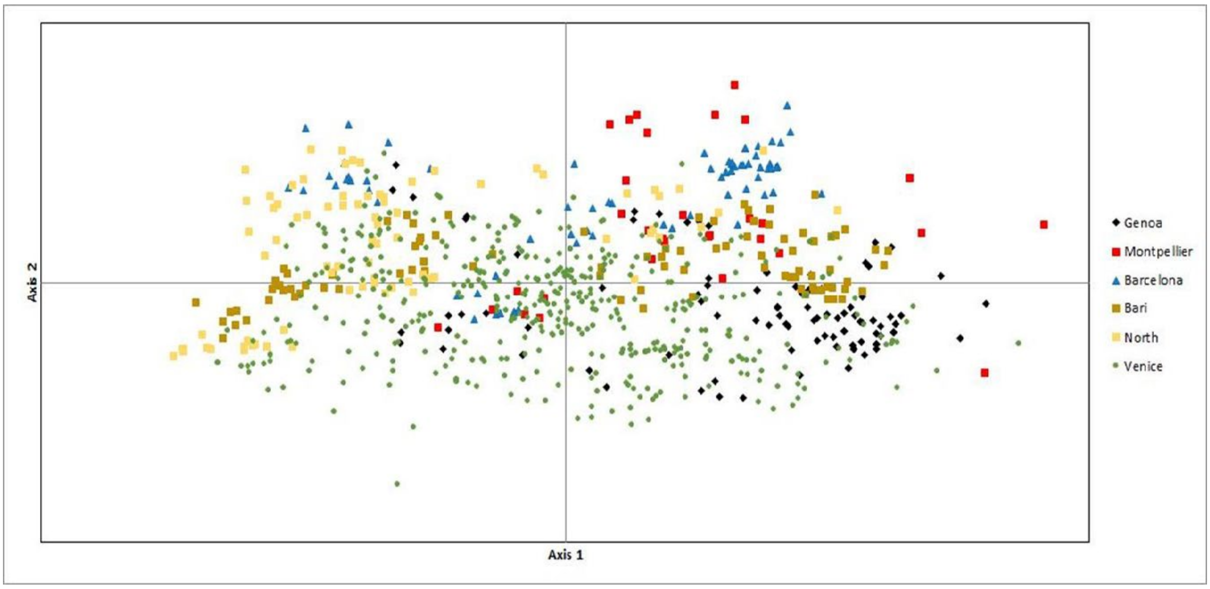

Supplementary Figure S5: Isolation by distance among populations within each of the six regions: **a**) Barcelona (R = 0.816), **b**) Montpellier (R = 0.517), **c**) Genoa (R = 0.250), **d**) Bari (R = 0.376), **e**) Venice (R = 0.134), and **f**) Northern Europe (R = 0.410); all R: $p < 0.001$. Geographical distance was rescaled into 10 distance classes (Fig. S6) with reference to the maximum pairwise distance of 60 populations (2187 km).

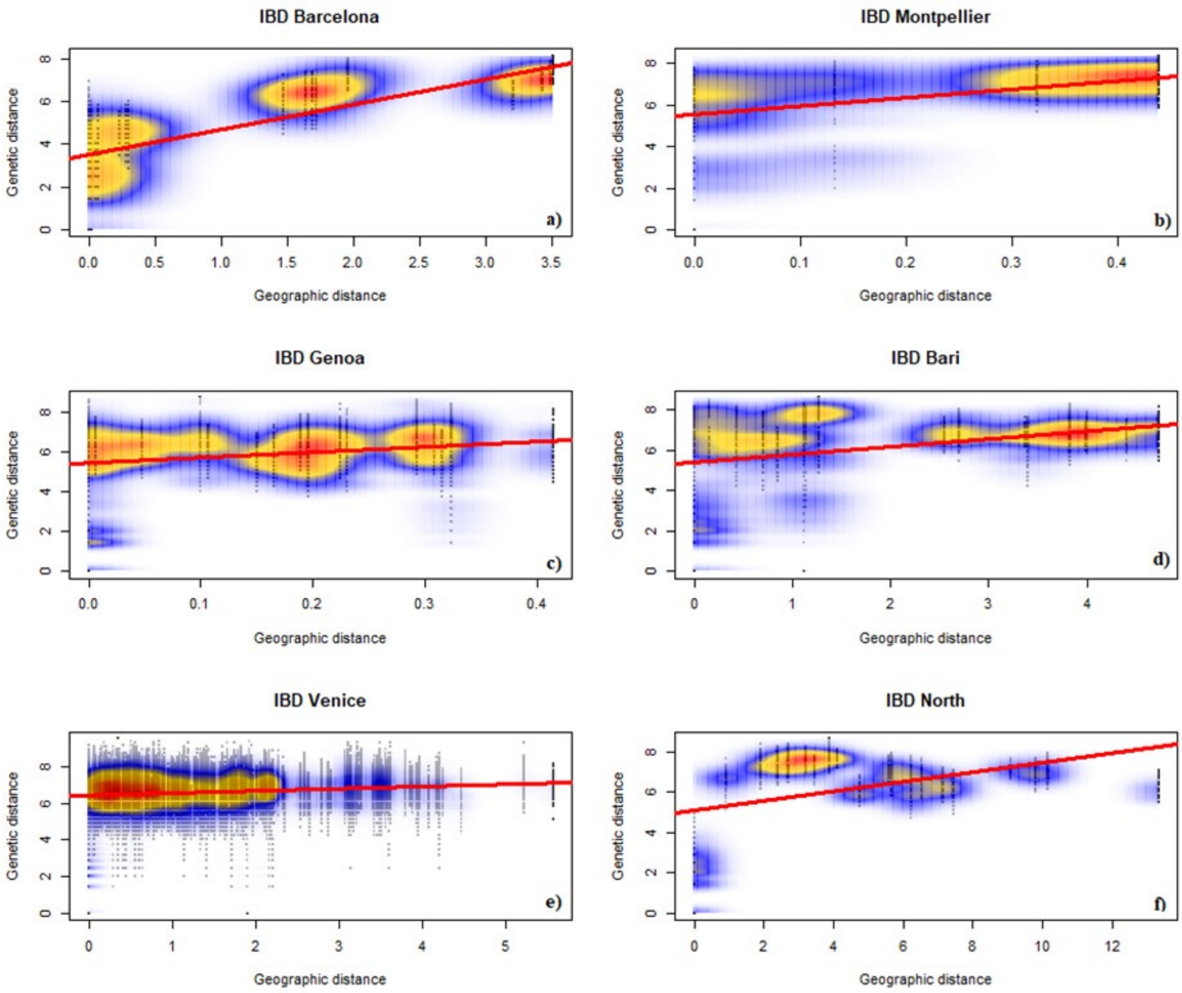

Supplementary Figure S6: Spatial autocorrelation analysis of 60 *Ambrosia psilostachya* populations showing the degree of non-random distribution of genotypes in space (geographical distance classes) based on random permutation and bootstrap estimates of r within the geographic distance of maximum 2187 km.

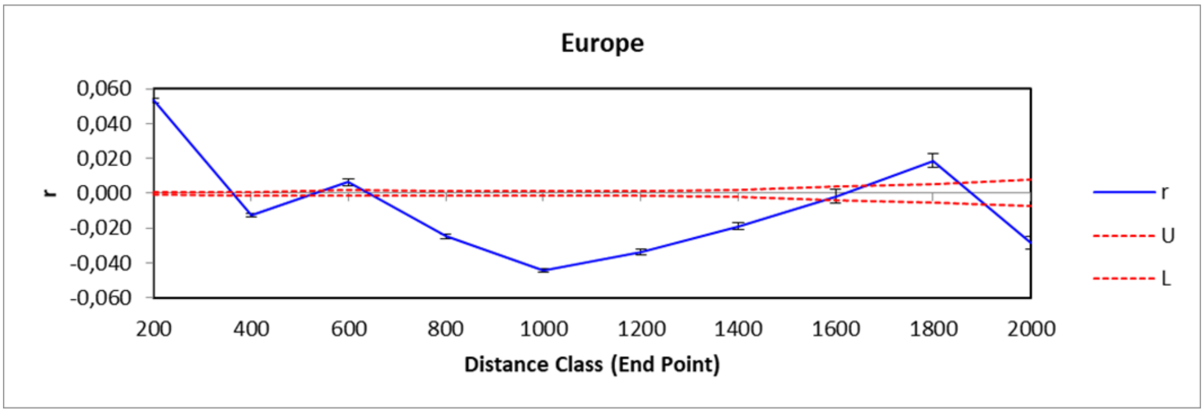

Supplementary Figure S7: Spatial autocorrelation analysis of *Ambrosia psilostachya* showing the degree of non-random distribution of genotypes in space (geographical distance classes) based on random permutation and bootstrap estimates of r tested for the six geographic regions: **a**) Barcelona, **b**) Montpellier, **c**) Genoa, **d**) Bari, **e**) Venice, and **f)** Northern Europe

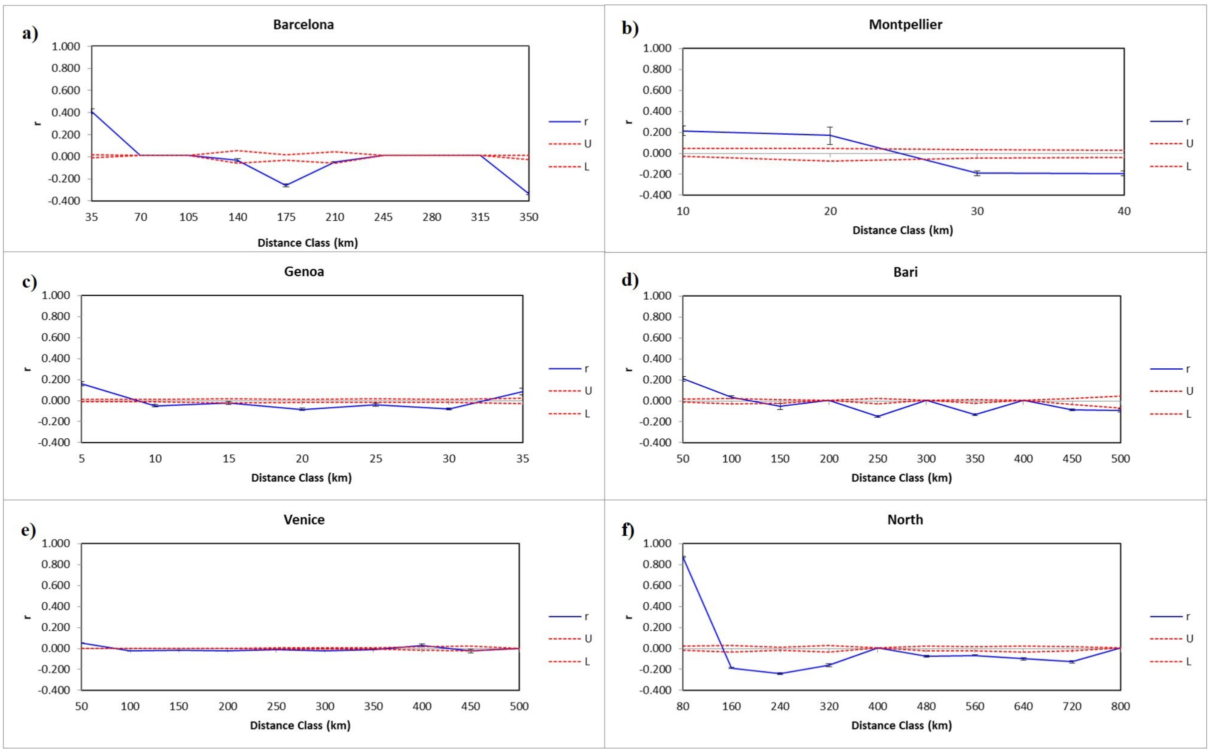

Supplementary Table S1: Means and standard deviation of population parameters and population genetic parameters of *Ambrosia psilostachya* from 60 European populations classified to 6 regions: size = total number of ramets per population, age = approximative age of the populations, n = number of ramets sampled, N = number of ramets genotyped per population, G/N = genet/ramet ratio; H = Shannon Index (based on N), E = Evenness, Na = allelic richness, H_o = observed heterozygosity, H_e = expected heterozygosity, and F_{is} = inbreeding coefficient; all calculations for the original and the clone corrected data set, after rarefaction.

region		size	age	n	N	G	G/N	H	E	Na cc	*Ho cc*	*He cc*	*F*is *cc*	Na ori	*Ho ori*	*He ori*	**F**is *ori*
Barcelona	mean	19183,33	51,67	15,83	15,33	11,33	0,78	2,32	0,88	1,99	0,53	0,37	-0,52	2,02	0,53	0,36	-0,53
	Std.-Dev.	11108,630	10,328	1,169	1,862	2,805	0,22	0,33	0,14	0,25	0,10	0,07	0,26	0,29	0,11	0,07	0,26
Bari	mean	7471,43	35,00	17,71	16,57	13,00	0,78	2,43	0,92	2,06	0,55	0,36	-0,33	2,13	0,55	0,36	-0,35
	Std.-Dev.	8787,202	23,979	2,059	2,637	4,899	0,24	0,51	0,08	0,55	0,11	0,14	0,36	0,64	0,11	0,14	0,37
Genoa	mean	31582,86	88,57	17,86	16,29	12,71	0,81	2,43	0,92	2,53	0,50	0,47	-0,18	2,63	0,50	0,46	-0,20
	Std.-Dev.	53023,058	17,008	3,934	4,271	3,773	0,21	0,34	0,08	0,47	0,10	0,07	0,32	0,56	0,10	0,07	0,34
Montpellier	mean	2366,67	103,33	12,00	10,67	10,33	0,98	2,29	0,99	2,98	0,45	0,53	0,02	3,21	0,45	0,52	0,01
	Std.-Dev.	2470,493	28,868	5,292	4,041	3,512	0,04	0,34	0,02	0,59	0,14	0,09	0,04	0,72	0,14	0,10	0,04
N Europe	mean	4875,00	102,50	15,88	15,25	9,88	0,67	2,08	0,81	1,67	0,40	0,25	-0,46	1,66	0,41	0,25	-0,49
	Std.-Dev.	2900,123	23,905	2,232	1,909	3,682	0,21	0,57	0,14	0,12	0,09	0,05	0,12	0,13	0,09	0,06	0,12
Venice	mean	48970,34	61,90	17,17	16,52	14,97	0,91	2,64	0,95	2,77	0,51	0,50	-0,04	2,95	0,51	0,50	-0,05
	Std.-Dev.	126596,909	6,184	3,060	2,911	3,375	0,12	0,28	0,08	0,53	0,09	0,10	0,23	0,64	0,10	0,11	0,23
total	mean	30912,00	68,33	16,75	15,92	13,20	0,84	2,47	0,92	2,45	0,50	0,44	-0,19	2,56	0,50	0,43	-0,20
	Std.-Dev.	91017,737	25,705	3,128	3,099	3,978	0,19	0,41	0,10	0,63	0,10	0,13	0,30	0,74	0,11	0,13	0,31

Supplementary Table S2: Clonal multiplication of *Ambrosia psilostachya* genets in 60 European populations: N = number of analyzed ramets per population, G = number of unique multilocus genotypes per population, nc = number of MLGs with clonal duplicates within the population, nd = number of clonal duplicates, clones_within = individual clones and their replicates within populations, clones_among = MLG duplicates among different populations

ID	location	N	G	nc	nd	clones_within					clones_among		
Psi3	Dépt. Gard, Saint-Laurent-d'Aigouze	10	10										
Psi4	Dépt. Gard, Aiques Mortes	7	7										
Psi5	Prov. Veneto, Alberoni	15	11	3	4	8/10	12/14/17	3/13					
Psi6	Prov. Veneto, Alberoni-Pellestrina	20	20										
Psi7	Prov. Veneto, Eraclea Mare, Laguna del Mort	18	17	1	1	5/9							
Psi8	Prov. Veneto, Eraclea Mare, Spiaggia del Mort	19	19								Psi8-14 = Psi9-6	Psi8-11 = 9-16	
Psi9	Mali Losinj, Ćunski	16	12	4	4	7/14	17/18	6/12	10/16		Psi9-6 = Psi8-14	Psi9-16 = 8-11	
Psi10	Prov. Veneto, Isola Verde 1	17	17										
Psi11	Prov. Veneto, Isola Verde 2	11	11										
Psi12	Prov. Veneto, Isola Verde 3	19	19										
Psi13	Prov. Veneto, Rosolina Mare 1	18	11	2	7	12/14/18/19/20	1/10/11/17						
Psi14	Prov. Veneto, Rosolina Mare 2	18	18										
Psi15	Prov. Veneto, Boccasette	18	16	2	2	7/8	18/20						
Psi16	Prov. Veneto, Barricata	15	15										
Psi17	Prov. Veneto, Porto Caleri	19	15	1	4	3/9/12/13/20							
Psi18	Prov. Emilia-Romagna, Lido di Volano 1	17	16	1	1	5/6							
Psi19	Prov. Emilia-Romagna, Lido di Volano 2	14	14										
Psi20	Prov. Emilia-Romagna, Lido di Spina	11	7	2	4	4/5/6	9/10/20						
Psi21	Prov. Tuskany, Marina di Massa	20	18	2	2	6/13	10/11						
Psi22	Prov. Tuskany, Forte dei Marmi 1	18	13	3	5	16/18/19	8/11	13/14/20					
Psi23	Prov. Tuskany, Forte dei Marmi 2	13	13										
Psi24	Prov. Tuskany, Torre del Lago	18	9	4	9	3/6/7	4/5/8/15	10/16/17/18	13/14				
Psi25	Prov. Tuskany, Marina di Torre del Lago	19	11	5	8	6/8	5/7	2/4/12/13/18	3/19	11/17			
Psi26	Prov. Tuskany, Bocca di Serchio	18	17	1	1	8/9							
Psi27	Prov. Tuskany, Cerasomma	8	8										
Psi28	Prov. Emilia-Romagna, Marina di Ravenna	20	18	2	2	2/4	16/17						
Psi29	Prov. Emilia-Romagna, Miramare di Rimini	18	18										
Psi30	Prov. Marken, Fosso Sejore	20	20										
Psi31	Prov. Marken, Senigallia	18	18										
Psi32	Prov. Abruzzi, Foro di Ortona 1	19	18	1	1	3/15					Psi32-19 = Psi34-11		
Psi33	Prov. Abruzzi, Foro di Ortona 2	15	5	5	10	10/14	9/13/15/16/17	3/11/12/19	4/5	1/7			
Psi34	Prov. Puglia, Lesina Marina	20	19	1	1	6/11					Psi34-11 = Psi32-19		
Psi35	Prov. Lombardia, Mezzanino	12	12										
Psi36	Prov. Emilia-Romagna, Quaresimo	19	19										
Psi37	Prov. Emilia-Romagna, Madregolo	20	15	3	5	4/9/14	6/19	10/11/13					
Psi38	Prov. Piedmont, Tortona	12	12										
Psi39	Prov. Veneto, Punta Sabbioni	12	12										
Psi40	Prov. Veneto, Vallevecchia	15	15										
Psi41	Prov. Emilia-Romagna, Lido di Classe	15	11	2	4	9/17/20	2/10/13						
Psi42	Prov. Emilia-Romagna, Lido di Dante	14	12	2	2	4/6	8/13						
Psi43	Dépt. Hérault, Pradez-le-Lez,	15	14	1	1	12/15							
Psi44	Catalonia, Barcelona, Platya El Prat	13	13										
Psi45	Catalonia, Barcelona, Montcada i Reixac	17	10	3	7	1/2/5/7/9/12	14/16	11/18					
Psi46	Catalonia, Barcelona, Martorelles	17	11	2	6	4/5/6/12/13/15	1/2				Psi46-17 = Psi47-14	Psi46-4/5/6/12/13/15 = Psi47-17	
Psi47	Catalonia, Barcelona, Montmelo	13	10	2	3	6/14/17	8/9				Psi47-14 = Psi46-17	Psi47-17 = Psi46-4/5/6/12/13/15	
Psi48	Valencia, L'Ampolla, Bassa de les Olles	16	16										
Psi49	Valencia, Tavernes de la Valldigna	16	8	5	8	12/17	2/7/8/20	4/10/14	3/11	15/18			
Psi50	Brandenburg, Hoyerswerda, Spreetal	19	16	2	3	7/9/13	3/14						
Psi51	Saxony-Anhalt, Grosskayna	14	13	1	1	1/6							
Psi52	Saxony-Anhalt, Gerwisch 1	13	6	4	7	5/13	8/9/10/17/20	14/15	1/18		Psi52-14/15 = Psi53-1	Psi52-8/9/10/17/20 = Psi53-15	Psi52-1/18 = Psi53-3/5/8/10/20
Psi53	Saxony-Anhalt, Gerwisch 2	16	9	3	7	3/5/8/10/20	12/19	7/1/15			Psi53-1 = Psi52-14/15	Psi53-15 = Psi52-8/9/10/17/20	Psi53-3/5/8/10/20 = Psi52-1/18
Psi54	Prov. North Holland, Ijmuiden	14	12	2	2	18/20	10/13						
Psi55	Prov. Halland, Falkenberg	16	8	3	8	9/14	6/7/11/13/16/20	3/4/17					
Psi56	West Pomerania, Szczecin	16	10	3	6	5/6/8/13/16	2/19	11/20					
Psi57	Kuyavian-Pomerania, Bydgoszcz	14	5	1	9	1/5/7/10/15/16/17/18/19/20							
Psi58	Prov. Puglia, Torre Mileto	17	14	3	3	16/20	3/13	15/17					
Psi59	Prov. Abruzzi, Porto Vasto	14	14										
Psi60	Prov. Calabria, Armondelea	13	9	2	4	6/8/11/14	10/18						
Psi61	Prov. Puglia, Punta Penna Grossa	18	12	3	6	8/16	10/11/19	3/5/9/20					
Psi62	Prov. Friuli-Venezia Giulia, Isola San Andrea	19	14	3	5	6/13/20	2/4/12	5/17					

Supplementary Table S3: First documented introductions of *Ambrosia psilostachya* to European countries based on herbarium specimens reviewed by the first author (Gerhard Karrer, voucher numbers were cited due to availability) and literature evaluation.

Country	year	source	status	source (lit., herbarium specimens)	comment
Albania	-	lit	-	lit: missing due to Raab-Straube & Raus (2016)	doubtful
Andorra	-	lit	-	lit: missing due to Coutinho & Paiva (2015)	
Austria	1952	specimen	casual	lit: Baschant (1955) was based on misidentification; but a specimen in herbarium RO: no 1931 was rev., by G. Karrer (Karrer 2021))	
Belarus	?	lit	?	lit: Eppo (2014)	doubtful
Belgium	1917	lit	naturalized	lit: Verloove (2006); oldest specimen 1918; Rompaey & Delvosalle 1972	
Bosnia Hercegovina	-	lit	-	no data in Raab-Straube & Raus (2016)	
Bulgaria	-	lit	-	lit: missing due to Raab-Straube & Raus (2016)	
Croatia	1932	specimen	naturalized	specimen: herbarium RO: Koludarc, 1932, misidentified as A. maritima (Karrer 2015), see also Lusina (1934), Melzer (1996)	
Czech Republic	1999	specimen	casual	lit: Červinka & Sádlo (2000), Pysek et al. (2012)	extinct?
Cyprus	-	lit	-	no data in Raab-Straube & Raus (2016)	
Denmark	1931	specimen	casual	specimen: LD 1457480, 1931, Herb Univ Copenhagen: Kulhusene	extinct?
Estonia	1933	specimen	casual	lit: Reier (2018, pers. comm)	extinct
Finland	2007	specimen	casual	specimen: private herbarium J. Särkkä, 2007;<br>lit: Lampinen & Lahti (2010)	
France	1891	specimen	naturalized	lit: Fried et al. (2015)	
Georgia	-	lit	-	no data in Raab-Straube & Raus (2016)	
Germany	1871	specimen	naturalized	specimens: 1894: Mannheim; 1911, Rüdersdorf, GZU: Nr. 259197 (1897-1927)	
Greece	2015	specimen	naturalized	lit: Raab-Straube & Raus (2016)	
Hungary	1962	lit	casual	lit: Soó (1970); personal observation Priszter (1962)	doubtful
Iceland	-	lit	-	no data in Raab-Straube & Raus (2016)	
Ireland	-	lit	-	lit: missing due to Rich (1994)	
Israel	2006	-	casual	lit: Yair et al. (2019), EPPO reporting service	extinct?
Italy	1924	specimen	naturalized	lit: Vignolo-Lutati (1935); specimen herbarium Pisa: Marina di Massa 1924, Sciapparo 1999, Armondelea 2013	
Kazakhstan	1970	lit	naturalized	lit: Buyankin (1975)	
Kosovo	-	lit	-	no data in Raab-Straube & Raus (2016)	
Latvia	1988	lit	casual	lit: Tabaka et al. (1988); Saar et al. (2000)	
Liechtenstein	-	-	casual	no source	doubtful
Lithuania	-	-	-	lit: missing due to Gudzinskas (1993)	
Luxembourg	1947	specimen	casual	specimen: herbarium LUX18796	extinct
Moldova	-	lit	-	lit: Karnkowski (2001) pest analysis Poland	doubtful
Montenegro	-	-	doubtful	lit: Pulević (2005)	
Netherlands	1905	specimen	naturalized	specimen herbarium L3421435, Nunspeed	
North-Macedonia	-	lit	-	lit: missing due to Raab-Straube & Raus (2016)	
Norway	1915	specimen	casual	specimen: Kristiansand, Kjøttkontrollen, 1915<br>misidentified specimen: Oslo (more recent)	extinct
Poland	1900	specimen	naturalized	specimen: herbarium S: S16-41153, Sczenczin, 1900 (was formerly misidentified as A. maritima; rev. G. Karrer 2016)	
Portugal	-	lit	-	lit: missing due to Coutinho & Paiva (2019)	
Romania	-	lit	-	misidentification by Ciocârlan & Constantin (1992); in consequence, also Sırbu (2012); revised by Karrer et al. (2021)	erroneous
Russia	1934	specimen	naturalized	specimen: herbarium UFA, Baschkortostan 1934; Herbarium Moskow State Univ. (MW) 1949; lit: Mosalenko (2001)	
Serbia	-	lit	-	no data in Raab-Straube & Raus (2016)	
Slovakia	-	lit	-	no data in Raab-Straube & Raus (2016)	
Slovenia	-	lit	-	no data in Raab-Straube & Raus (2016)	
Spain	1947	specimen	naturalized	lit: Lawalree (1947); specimens: herbarium BCN: Mollet:1955; Montacado: 1961, L'Ampollea: 1973, Tavernes de Valdigna: 1982	
Sweden	1911	specimen	naturalized	lit: Karlsson (1998); specimen: LD-1456220 Åhus, 1911, SN0541-3593, Klagshamn, 1931; PL02443358, Klagsdorp, 1933	
Switzerland	1922	specimen	casual	lit: Hegi (1979): Hohtenn, Wallis, 1922; Lawalree (1947: Ascona, 1946); specimen GZU: 000230745, 1939, Basel	
Turkey	-	lit	-	lit: missing due to Raab-Straube & Raus (2016)	
Ukraine	1967	specimen	naturalized	specimen: herbarium MW 9146; lit: Mosyakin & Fedoronchuk (1999)	
United Kingdom	1880	lit	naturalized	lit: Rich (1994)	

Literature used to fit Supplementary Table S3:

Baschant, R. Ruderalflächen und deren Pflanzen in und um Linz. *Naturk. Jahrb. Stadt Linz 1955*, 253–261 (1955).

Buyankin, V. I. New weeds of the Ural'sk Province. *Botanicheskii Zhurn.* **60**, 1190–1191 (1975).

Ciocârlan, V. & Constantin, P. O. Nouă specie adventivă în flora României: *Ambrosia coronopifolia* Torrey et A. Gray. *Analele Ştiinţifice ale Institutului Delta Dunării 1992*, 49–50 (1992).

Červinka, Z. & Sádlo, J. Neofyty Ambrosia psilostachya a Celastrus orbiculatus v městě Čelákovicích. *Muzeum a současnost, ser. natur.* **14**, 65–68 (2000).

Coutinho, A. P. & Paiva, J. *Ambrosia.* in *Flora-Peninsula Iberica* Vol XVI(III) (Ed. Benedi, C. et al.) 2150–2156. (Real Jardin Botanico Madrid, 2019).

EPPO Plants regulated as quarantine pests in Belarus. *EPPO Reporting service* **2014/041** (2014).

Fried, G., Chauvel, B., Reynaud, P. & Sache, I. Decreases in crop production by non-native weeds, pests, and pathogens in *Impact of biological invasions on ecosystem services* Vilà, M. (ed.) 83–101 (Springer, 2017).

Gudzinskas, Z. Genus *Ambrosia* (L.) (*Asteraceae*) in Lithuania. *Thaiszia* **3**, 89–96 (1993).

Karlsson, T. Förteckning över svenska kärlväxter. *Svensk botanisk tidskrift* **91,** 241–560 (1998).

Karnkowski, W. *Pest Risk Analysis and Pest Risk Assessment for the territory of the Republic of Poland (as PRA area) on Ambrosia spp., updated version*. (Torun, 2001). https://www.eppo.int/QUARANTINE/Pest_Risk_Analysis/PRAdocs_plants/08-14124%20PRA-Ambrosia.doc

Karrer, G. *Ambrosia coronopifolia* Torr, et A. Gray distribution. in *Flora Croatica Database* (Nikolic, T., http://hirc.bofanic.hr/fcd). (Faculty of Science, University of Zagreb, 2015) (accessed 01 Dec 1918).

Karrer, G. Interessante Gefäßpflanzen-Funde aus Österreich, 1. *Neilreichia* **12**, 183–187 (2021).

Karrer, G. et al. *Ambrosia tenuifolia*, instead of *A. psilostachya*, in Romania. *Scientific Annals of the Danube Delta Institute* **26**, 17–26 (2021).

Lampinen, R. & Lahti, T. *Kasviatlas 2009*. (Helsingin Yliopisto, Luonnontieteellinen keskusmuseo, Kasvimuseo, Helsinki. Levinneisyyskartat osoitteessa, 2010) http://www.luomus.fi/kasviatlas.

Lawalreé, A. Les Ambrosia adventices en Europe occidentale. *Bull. Jard. Botan. l'Etat Bruxelles* **18**, 305–315 (1947).

Lusina, G. Escursioni botaniche su alcune isole minori del Carnaro. *Boll. Soc. Adriat. Sci. Nat. Trieste* **33**, 27-65 (1934).

Melzer, H. Neues zur Flora von Slowenien und Kroatien. *Hladnikia* **7**, 5–10 (1996).

Moskalenko, G. P. Ambrosia psilostachya in: *Zashchita i Karantin Rasteniĭ (Invasive species compendium)*, No.3, 36–37. (Izdatel'stvo Kolos, Moscow, 2002).

Mosyakin, S. L. & Fedoronchuk, M. M. *Vascular plants of Ukraine. A nomenclatural checklist*. (M. G. Kholodny Institute of Botany, National Academy of Sciences of Ukraine Kiev, 1999).

Pulević, V. *Građa za vaskularnu floru Crne Gore Podgorica*. (Podgorica, 2005).

Pyšek, P., Chytrý, M., Pergl, J., Sádlo, J. & Wild, J. Plant invasions in the Czech Republic: current state, introduction dynamics, invasive species and invaded habitats. *Preslia* **84**, 575–629 (2012).

Rich, T. C. G. Ragweeds (*Ambrosia* L.) in Britain. *Grana* **33**, 38–43 (1994).

Saar, M. et al. Ragweed plants and airborne pollen in the Baltic states. *Aerobiologia* **16**, 101–106 (2000).

Sırbu, C. *Plante de carantina invazive ın Romania*. (Bukarest, 2012).

Soó, R. *A magyar flóra és vegetáció rendszertani-növényföldrajzi kézikönyve IV.* (Akadémiai Kiadó, Budapest, 1970).

Tabaka, L., Gavrilova, G. & Fatare, I. *Flora of Vascular Plants of the Latvian SSR* (in Russian). (Zinatne, Riga, 1988).

Van Rompaey, E. & Delvosalle, L. *Atlas van de Belgische en Luxemburgse flora – Pteridofyten en Spermatofyten*. (Meise, 1972).

Verloove, F. Catalogue of neophytes in Belgium (1800-2005). *Scripta Botanica Belgica* **39**, 1–89 (2006).

Vignolo-Lutati, F. Il Genere "*Ambrosia*„ in Italia. *Giorn. botan. ital.* **42**, 364–378 (1935).

Von Raab-Straube E. & Raus T. (eds.) Euro+Med-Checklist Notulae 6. *Willdenowia* **46**, 423–442 (2016).

Wagenitz, G. (ed.) *Illustrierte Flora von Mitteleuropa. Compositae I: Allgemeiner Teil, Eupatorium – Achillea. 2. Auflage* (München, 1979).

Yair, Y. et al. Ragweed species (*Ambrosia* spp.) in Israel: distribution and allergenicity. *Aerobiologia* **35**, 85–95 (2019).

Supplementary Table S4: Origin and voucher number (Herbarium WHB) of *Ambrosia psilostachya* populations sampled from Europe in 2014-2017.

ID	Country	Region	Location (province, town)	Latitude	Longitude	Year	WHB number
Psi-03	France	Montpellier	Dépt Gard, Saint-Laurent-d'Aigouze	43.576191	4.283959	2014	80982
Psi-04	France	Montpellier	Dépt Gard, Aiques Mortes	43.560046	4.152070	2014	80983
Psi-05	Italy	Venice	Prov Veneto, Alberoni	45.340992	12.327727	2015	80984
Psi-06	Italy	Venice	Prov Veneto, Alberoni-Pellestrina	45.339774	12.327115	2015	80985
Psi-07	Italy	Venice	Prov Veneto, Eraclea Mare, Laguna del Mort	45.539494	12.743016	2015	80986
Psi-08	Italy	Venice	Prov Veneto, Eraclea Mare, Spiaggia del Mort	45.531963	12.735872	2015	80987
Psi-09	Croatia	Venice	Mali Losinj, Ćunski	44.585083	14.399433	2015	80988
Psi-10	Italy	Venice	Prov Veneto, Isola Verde 1	45.177470	12.319835	2016	80989
Psi-11	Italy	Venice	Prov Veneto, Isola Verde 2	45.177689	12.319632	2016	80990
Psi-12	Italy	Venice	Prov Veneto, Isola Verde 3	45.178514	12.319707	2016	80991
Psi-13	Italy	Venice	Prov Veneto, Rosolina Mare 1	45.112992	12.325877	2016	80992
Psi-14	Italy	Venice	Prov Veneto, Rosolina Mare 2	45.112616	12.328462	2016	80993
Psi-15	Italy	Venice	Prov Veneto, Boccasette	45.026543	12.424633	2016	80994
Psi-16	Italy	Venice	Prov Veneto, Barricata	44.852682	12.469125	2016	80995
Psi-17	Italy	Venice	Prov Veneto, Porto Caleri	45.095918	12.328046	2016	80996
Psi-18	Italy	Venice	Prov Emilia-Romagna, Lido di Volano 1	44.803685	12.266516	2016	80997
Psi-19	Italy	Venice	Prov Emilia-Romagna, Lido di Volano 2	44.803520	12.266661	2016	80998
Psi-20	Italy	Venice	Prov Emilia-Romagna, Lido di Spina	44.646999	12.250766	2016	80999
Psi-21	Italy	Genoa	Prov Tuskany, Marina di Massa	44.029340	10.060986	2016	81000
Psi-22	Italy	Genoa	Prov Tuskany, Forte dei Marmi 1	43.976064	10.144794	2016	81001
Psi-23	Italy	Genoa	Prov Tuskany, Forte dei Marmi 2	43.972090	10.150423	2016	81002
Psi-24	Italy	Genoa	Prov Tuskany, Torre del Lago	43.829812	10.275942	2016	81003
Psi-25	Italy	Genoa	Prov Tuskany, Marina di Torre del Lago	43.816077	10.260793	2016	81004
Psi-26	Italy	Genoa	Prov Tuskany, Bocca di Serchio	43.782051	10.269448	2016	81005
Psi-27	Italy	Genoa	Prov Tuskany, Cerasomma	43.834382	10.426395	2016	81006
Psi-28	Italy	Venice	Prov Emilia-Romagna, Marina di Ravenna	44.470872	12.285299	2016	81007
Psi-29	Italy	Venice	Prov Emilia-Romagna, Miramare di Rimini	44.025005	12.630965	2016	81008
Psi-30	Italy	Venice	Prov Marken, Fosso Sejore	43.878346	12.966665	2016	81009
Psi-31	Italy	Venice	Prov Marken, Senigallia	43.748620	13.174151	2016	81010
Psi-32	Italy	Bari	Prov Abruzzi, Foro di Ortona 1	42.397488	14.334697	2016	81011
Psi-33	Italy	Bari	Prov Abruzzi, Foro di Ortona 2	42.398241	14.332099	2016	81012
Psi-34	Italy	Bari	Prov Puglia, Lesina Marina	41.906049	15.352998	2016	81013
Psi-35	Italy	Venice	Prov Lombardia, Mezzanino	45.126077	9.205948	2016	81014
Psi-36	Italy	Venice	Prov Emilia-Romagna, Quaresimo	44.688881	10.551157	2016	81015
Psi-37	Italy	Venice	Prov Emilia-Romagna, Madregolo	44.797190	10.205036	2016	81016
Psi-38	Italy	Venice	Prov Piedmont, Tortona	44.898117	8.846820	2016	81017
Psi-39	Italy	Venice	Prov Veneto, Punta Sabbioni	45.622370	12.960443	2017	81018
Psi-40	Italy	Venice	Prov Veneto, Vallevecchia	45.622156	12.959085	2017	81019
Psi-41	Italy	Venice	Prov Emilia-Romagna, Lido di Classe	44.330598	12.335721	2017	81020

Psi-42	Italy	Venice	Prov Emilia-Romagna, Lido di Dante	44.389205	12.317627	2017	81021
Psi-43	France	Montpellier	Dépt Hérault, Pradez-le-Lez,	43.719511	3.869237	2017	81022
Psi-44	Spain	Barcelona	Catalonia, Barcelona, Platya El Prat	41.283398	2.093170	2017	81023
Psi-45	Spain	Barcelona	Catalonia, Barcelona, Montcada i Reixac	41.493499	2.192812	2017	81024
Psi-46	Spain	Barcelona	Catalonia, Barcelona, Martorelles	41.535985	2.229382	2017	81025
Psi-47	Spain	Barcelona	Catalonia, Barcelona, Montmelo	41.546996	2.250274	2017	81026
Psi-48	Spain	Barcelona	Valencia, L'Ampolla, Bassa de les Olles	40.788987	0.706413	2017	81027
Psi-49	Spain	Barcelona	Valencia, Tavernes de la Valldigna	39.076306	-0.242532	2017	81028
Psi-50	Germany	N Europe	Brandenburg, Hoyerswerda, Spreetal	51.491255	14.354334	2017	81029
Psi-51	Germany	N Europe	Saxony-Anhalt, Grosskayna	51.292169	11.950130	2017	81030
Psi-52	Germany	N Europe	Saxony-Anhalt, Gerwisch 1	52.193148	11.736143	2017	81031
Psi-53	Germany	N Europe	Saxony-Anhalt, Gerwisch 2	52.191249	11.736525	2017	81032
Psi-54	Netherlands	N Europe	Prov North Holland, Ijmuiden	52.452215	4.573574	2017	81033
Psi-55	Sweden	N Europe	Prov Halland, Falkenberg	56.873796	12.514821	2017	81034
Psi-56	Poland	N Europe	West Pomerania, Szczecin	53.397667	14.689132	2017	81035
Psi-57	Poland	N Europe	Kuyavian-Pomerania, Bydgoszcz	53.155929	17.891963	2017	81036
Psi-58	Italy	Bari	Prov Puglia, Torre Mileto	41.907821	15.511118	2017	81037
Psi-59	Italy	Bari	Prov Abruzzi, Porto Vasto	42.172938	14.703322	2017	81038
Psi-60	Italy	Bari	Prov Calabria, Armondelea	37.931175	15.889648	2017	81039
Psi-61	Italy	Bari	Prov Puglia, Punta Penna Grossa	40.720647	17.773990	2017	81040
Psi-62	Italy	Venice	Prov Friuli-Venezia Giulia, Isola San Andrea	45.716307	13.237508	2017	81041

Supplementary Table S5: Nuclear DNA contents of *Ambrosia psilostachya* populations from Europe; origin of populations, standard used, standard size (pg), CV = coefficient of variation, mean sample value (n=3; pg).

Relative genome size of *A. psilostachya* (n =10) specimens were measured by flow cytometry using the CyStain PI Absolute P kit (Sysmex, Görlitz, Germany) according to the manufacturer's manual using leaf tissue from the sample together with an internal standard: *Solanum nigrum* (=2.6 pg) and *Pisum sativum* (= 8.8 pg). In a CyFlow Space (532 nm diode laser; Sysmex) the nuclear DNA content was measured for each sample until at least 5000 counts were obtained.

Origin	Standard	Standard size (pg)	CV (%)	Mean sample value (pg)
Barcelona Platya El Prat (Psi-44)	*Pisum sativum*	8.8	3.08	5.4
Montcada i Reixac (Psi-45)	*Solanum nigrum*	2.6	2.98	5.2
Rosolina Mare (Psi-13)	*Solanum nigrum*	2.6	2.37	5.2
Punta Sabbioni (Psi-39)	*Solanum nigrum*	2.6	3.98	5.4
Vallevecchia (Psi-40)	*Solanum nigrum*	2.6	3.56	5.4
Lido di Classe (Psi-41)	*Solanum nigrum*	2.6	2.76	5.3
Lido di Dante (Psi-42)	*Solanum nigrum*	2.6	1.97	5.2
Foce Schiapparo (Psi-58)	*Solanum nigrum*	2.6	2.21	5.3
Punta Penna Grossa (Psi-59)	*Solanum nigrum*	2.6	2.29	5.2
Castagna Vecchia, Teramo (Psi-87)	*Pisum sativum*	8.8	2.79	5.4
